# Effects of a personalized nutrition program on cardiometabolic health: a randomized controlled trial

**DOI:** 10.1038/s41591-024-02951-6

**Published:** 2024-05-08

**Authors:** Kate M. Bermingham, Inbar Linenberg, Lorenzo Polidori, Francesco Asnicar, Alberto Arrè, Jonathan Wolf, Fatema Badri, Hannah Bernard, Joan Capdevila, William J. Bulsiewicz, Christopher D. Gardner, Jose M. Ordovas, Richard Davies, George Hadjigeorgiou, Wendy L. Hall, Linda M. Delahanty, Ana M. Valdes, Nicola Segata, Tim D. Spector, Sarah E. Berry

**Affiliations:** 1 Department of Nutritional Sciences, King’s College London, London, UK; 2 Zoe Ltd, London, UK; 3 Department of Cellular, Computational and Integrative Biology, University of Trento, Trento, Italy; 4 Emory University School of Medicine, Atlanta, GA USA; 5 Stanford University, Stanford, CA USA; 6 Jean Mayer USDA Human Nutrition Research Center on Aging, Tufts University, Boston, MA USA; 7 IMDEA Food Institute, Campus of International Excellence, Universidad Autónoma de Madrid, Consejo Superior de Investigaciones Científicas, Madrid, Spain; 8 Universidad Camilo José Cela, Madrid, Spain; 9 Diabetes Center, Massachusetts General Hospital and Harvard Medical School, Boston, MA USA; 10 School of Medicine, University of Nottingham, Nottingham, UK; 11 Nottingham National Institute for Health and Care Research Biomedical Research Centre, Nottingham, UK; 12 Department of Twin Research and Genetic Epidemiology, King’s College London, London, UK

**Keywords:** Disease prevention, Machine learning

## Abstract

Large variability exists in people’s responses to foods. However, the efficacy of personalized dietary advice for health remains understudied. We compared a personalized dietary program (PDP) versus general advice (control) on cardiometabolic health using a randomized clinical trial. The PDP used food characteristics, individual postprandial glucose and triglyceride (TG) responses to foods, microbiomes and health history, to produce personalized food scores in an 18-week app-based program. The control group received standard care dietary advice (US Department of Agriculture Guidelines for Americans, 2020–2025) using online resources, check-ins, video lessons and a leaflet. Primary outcomes were serum low-density lipoprotein cholesterol and TG concentrations at baseline and at 18 weeks. Participants (*n* = 347), aged 41–70 years and generally representative of the average US population, were randomized to the PDP (*n* = 177) or control (*n* = 170). Intention-to-treat analysis (*n* = 347) between groups showed significant reduction in TGs (mean difference = −0.13 mmol l^−1^; log-transformed 95% confidence interval = −0.07 to −0.01, *P* = 0.016). Changes in low-density lipoprotein cholesterol were not significant. There were improvements in secondary outcomes, including body weight, waist circumference, HbA1c, diet quality and microbiome (beta-diversity) (*P* < 0.05), particularly in highly adherent PDP participants. However, blood pressure, insulin, glucose, C-peptide, apolipoprotein A1 and B, and postprandial TGs did not differ between groups. No serious intervention-related adverse events were reported. Following a personalized diet led to some improvements in cardiometabolic health compared to standard dietary advice. ClinicalTrials.gov registration: NCT05273268.

## Abstract (web summary)

A randomized controlled trial showed that following a personalized dietary program led to significant improvements in cardiometabolic and gut health as well as reductions in body weight compared to following standard dietary advice according to US Department of Agriculture guidelines.

## Main

Chronic diseases underpinned by diet and lifestyle exposures are among the leading causes of death globally. Diet and lifestyle strategies can be an effective approach to reduce risk for many chronic diseases^1,2^. However, despite evidence for the effectiveness of such approaches, rates of diet-related diseases continue to increase. This may in part be due to poor adherence to population guidelines and because of the large variability in how people respond to foods^3,4^, such that a single dietary approach is not the most effective for everyone. Indeed, in the United States, adherence to the Dietary Guidelines for Americans is well below the recommended levels for health^5^; less than 1% of UK individuals follow all core nine dietary recommendations^6^. Furthermore, we now know the large intraindividual and interindividual variability observed in individual health responses to food are associated with multiple factors^3^. Therefore, personalized nutrition programs that are based on biological, phenotypic and lifestyle advice offer promise to improve both adherence and efficacy.

Observational research supports the application of personalized nutrition^7,8^ but there are few randomized controlled trials designed to test the efficacy of personalized nutrition programs compared to standard dietary advice on health outcomes. Overall, dietary quality is improved by personalized nutrition programs tailored on baseline dietary information, phenotypic, genotypic or lifestyle factors, compared to nonpersonalized advice^9^. Personalization of dietary advice can assist and motivate individuals to follow a healthier diet and lifestyle^10^. Furthermore, a personalized diet integrating glycemic response, blood parameters, dietary habits, anthropometrics, physical activity and gut microbiota, resulted in greater improvements in markers of glycemic and lipemic control compared to a Mediterranean diet^11^. Personalized nutrition approaches and corresponding studies typically use a single axis of personalization but reported low correlations between biomarkers, for example, triglycerides (TGs) and glucose, suggesting that a prediction algorithm using a multilevel approach to personalization may yield superior results.

**Fig. 1:**
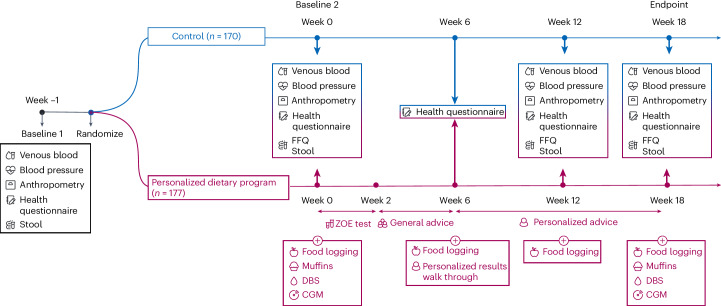
METHOD study design. *n* = 177 participants were allocated to the PDP intervention group and *n* = 170 participants were allocated to the control group. DBS, dried blood spot finger-prick test. CGM, continuous glucose monitor. A Food Frequency Questionnaire (FFQ), accompanied by a dietary behavior survey, was administered. Anthropometry measures included waist circumference, hip circumference, height and body weight.

Therefore, we hypothesized that a multilevel approach to personalization encompassing multiple factors contributing to intraindividual and interindividual variability in nutritional responses to diet will improve the efficacy of advice to elicit a meaningful impact on health outcomes. This 18-week randomized controlled trial (the ZOE Measuring Efficacy THrough Outcomes of Diet (METHOD) study) assessed a personalized dietary program (PDP) underpinned by multiple biological inputs (glucose, TGs, microbiome and health history) and overlaid with generalized dietary and lifestyle advice (Fig. 1) versus the United States Department of Agriculture (USDA) recommended diet (control) on cardiometabolic risk and microbiome composition in a generally representative adult US population.

## Results

### Study participant disposition

**Fig. 2:**
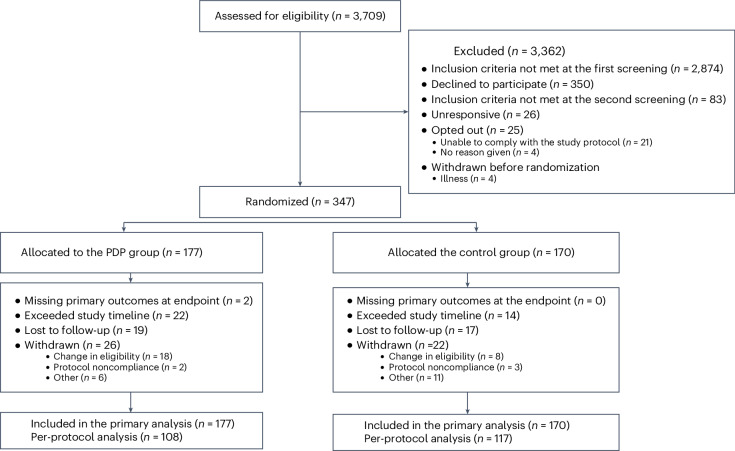
CONSORT diagram. CONSORT, Consolidated Standards of Reporting Trials.

Between 1 March 2022 and 10 August 2022, 3,709 participants were screened for enrollment; 347 participants were randomly assigned to the PDP (*n* = 177) or control (*n* = 170) group and were included in the full analysis set (all randomized participants according to the intention-to-treat (ITT) principle). Of the 347 participants, *n* = 225 were included in the per-protocol analysis. Recruitment, randomization and follow-up numbers are summarized in the Consolidated Standards of Reporting Trials (CONSORT) diagram in Fig. 2.

Participant characteristics are shown in Table 1 and were similar between groups at baseline. In total, 86% of participants were female and had a mean ± s.d. age of 52 ± 7.5 years, body mass index (BMI) of 34 ± 5.8 kg m^−^^2^, fasting serum glucose of 5.32 mmol l^−1^ (95% confidence interval (CI) = 5.25 to 5.40), fasting total cholesterol of 5.41 mmol l^−1^ (95% CI = 5.32 to 5.49), TG concentrations of 1.35 mmol l^−1^ (95% CI = 1.29 to 1.41) and low-density lipoprotein cholesterol (LDL-C) concentrations of 3.37 mmol l^−1^ (95% CI = 3.30 to 3.44). Compared to a US representative population (National Health and Nutrition Examination Survey 2017–2018), ZOE METHOD study participants had a similar waist circumference (104 cm versus 101 cm); in similarly aged individuals (40.0–69.9 years) they had a slightly higher BMI (BMI > 30 kg m^−^^2^; 52% versus 41%)^12^.

**Table 1:** Descriptive characteristics of the participants^a^

	Total (*n* = 347)	*n*	PDP (*n* = 177)	*n*	Control (*n* = 170)	*n*
Sex, male/female (*n*)	47/300	347	27/150	177	20/150	170
Age (years)	52 (7.5)	345	52 (7.8)	177	52 (7.1)	170
Menopausal status (%)						
Premenopausal	22	67	21	32	23	35
Perimenopausal	26	79	25	38	27	41
Postmenopausal	40	120	45	67	35	53
Unknown	11	34	9	13	14	21
Ethnicity (%)						
American Indian or Alaskan Native	0.3	1	1	1	0	0
Asian	5.8	20	6	10	6	10
Black or African American	4.3	15	4	7	5	8
Hispanic	1.7	6	2	4	1	2
Multiracial	0.6	2	1	2	0	0
White	82	285	82	146	82	139
Unknown	5	18	4	7	6	11
Physical activity status (%)						
Less than once per month	17	60	19	34	15	26
Once a week	16	55	12	22	19	33
Twice a week	23	80	24	42	22	38
Three to four times a week	27	94	27	47	28	47
Five or more	11	39	14	24	9	15
Unknown	5	19	5	8	6	11
Education status (%)						
High school diploma or equivalent	13	44	12	22	13	22
More than one degree	41	143	41	72	42	71
One degree	39	134	41	72	36	62
Other	2	6	2	4	1	2
Unknown	6	20	4	7	8	13
BMI (kg m^−^^2^)	33.6 (5.8)	341	33.1 (5.52)	175	34.1 (6.04)	166
Waist circumference (cm)	104 (12.1)	345	104 (12.1)	176	104 (12.1)	169
Systolic blood pressure (mmHg)	124 (16.4)	344	123 (16.0)	176	126 (16.9)	168
Diastolic blood pressure (mmHg)	79.7 (10.8)	344	79.1 (10.6)	176	80.3 (11.1)	168
Glucose (mmol l^−1^)^b^	5.32 (5.24 to 5.40)	347	5.24 (5.16 to 5.33)	177	5.40 (5.27 to 5.54)	170
HbA1c (%)^b^	5.39 (5.35 to 5.44)	345	5.34 (5.30 to 5.39)	176	5.45 (5.36 to 5.54)	169
Total cholesterol (mmol l^−1^)	5.41 (5.32 to 5.49)	347	5.34 (5.24 to 5.45)	177	5.47 (5.34 to 5.60)	170
TGs (mmol l^−1^)^b^	1.35 (1.29 to 1.41)	347	1.33 (1.26 to 1.42)	177	1.37 (1.29 to 1.46)	170
Low-density lipoprotein cholesterol (mmol l^−1^)	3.38 (3.30 to 3.45)	346	3.29 (3.19 to 3.39)	176	3.46 (3.35 to 3.57)	170
High-density lipoprotein cholesterol (mmol l^−1^)	1.53 (1.50 to 1.57)	347	1.57 (1.52 to 1.62)	177	1.50 (1.46 to 1.55)	170
HEI score (0–100)	66.8 (9.07)	277	65.6 (10.3)	134	67.9 (7.61)	143

Data are mean ± s.d. or mean (95% CI), unless indicated otherwise. ^a^No significant differences between groups, except for the HEI score: two-sample test. ^b^Geometric mean and 95% CIs presented.

### Dietary intake

**Fig. 3:**
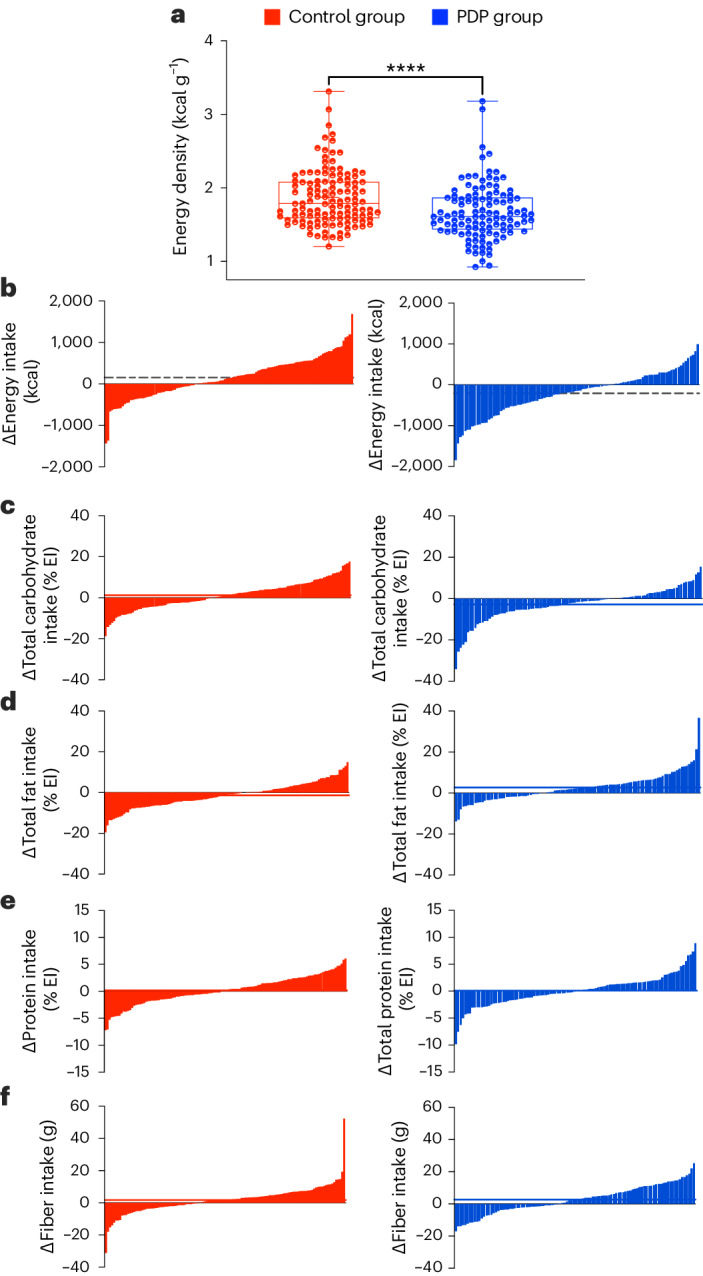
Dietary intake. **a**, Mean energy density (kcal g^−1^) of the diet at the study endpoint for the control group (red) (*n* = 120 participants with dietary data available) and PDP group (blue) (*n* = 111 participants with dietary data available). An unpaired, two-sided, between-group *t*-test was used (*P* < 0.001). Data presented include the first quartile, median and third quartile. **b**–**f**, Individual change in energy and nutrient intake before and after the intervention for energy intake (kcal) (**b**), carbohydrate (% EI) (**c**), fat (% EI) (**d**), protein (% EI) (**e**) and fiber (g) (**f**) intake across the control (red) and PDP (blue) groups.

The composition of the participants’ habitual diets at baseline is shown in Supplementary Table 1. Participants in both groups had a mean (95% CI) change in energy intake from baseline, with the PDP group reducing energy intake versus the control group (mean difference in change between groups 162 kcal per day (95% CI = 22.0 to 302), *P* < 0.001 for the interaction between diet group and time, adjusted for age and sex). In the PDP versus control diet, the mean 18-week macronutrient distributions were 39% versus 41% for carbohydrates, 46% versus 44% for fat and 16% versus 16% for protein. There were significant between-group differences at week 18 (all *P* ≤ 0.05) for the percentage of energy from carbohydrates, fat, polyunsaturated fatty acids, fiber and energy density (Supplementary Table 1). The PDP was a lower energy density diet compared to the control at week 18 (mean ± s.d., 1.67 ± 0.38 versus 1.87 ± 0.38 kcal g^−1^, *P* < 0.001) (Fig. 3a).

To demonstrate interindividual variability in dietary intake achieved through personalized and general advice, we assessed the variability in nutrient and food intake. The individual changes in energy and nutrient intake before and after intervention were highly variable between participants following both interventions (Fig. 3b–f). There was also large variability for individual foods and food groups at the study endpoint, as measured using the coefficient of variation (CV), following the control (mean CV = 262%) and PDP (mean CV = 248%).

### Adherence to the advice

Participants in both interventions were asked to self-report adherence to the dietary advice using a questionnaire; 30% more participants reported high or very high subjective adherence (scores ≥ 8, respectively, on a 0–10 scale) to the dietary advice in the PDP versus control group. Participants in both the PDP and control groups with the greatest achieved improvement in overall diet quality (top 30th percentile of the Healthy Eating Index (HEI) score) increased diet quality by 12.9% (mean ± s.d.; 8.41 ± 8.47) and 6.15% (4.0 ± 6.59), respectively. In the PDP group, adherence to the program was also assessed through logging metrics and personalized day scores derived from logged diet data (Supplementary Table 2).

### Primary outcomes

**Fig. 4:**
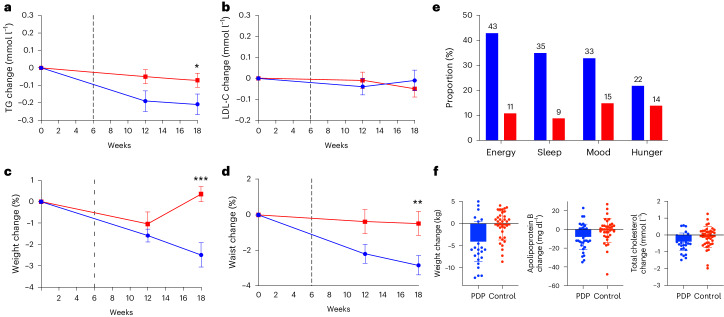
Changes in primary and selected secondary outcomes during the intervention period. **a**–**d**, Mean ± s.e.m. changes from baseline values in TG (mmol l^−1^) (*P* = 0.016) (**a**), LDL-C (mmol l^−1^) (**b**), weight (%) (*P* < 0.001) (**c**) and waist circumference (%) (*P* = 0.008) (**d**), in participants allocated to the PDP (blue line) (*n* = 177) or control (red line) (*n* = 172) group. Repeated measures model between groups. **e**, Proportion (%) of participants in the PDP and control groups with subjective improvements in energy level, sleep quality, mood, and hunger levels. **f**, Changes in weight (kg), apolipoprotein B (mg dl^−1^) and total cholesterol (mmol l^−1^) for highly adherent PDP (*n* = 35) and controls (*n* = 39) (mean and s.e.m. shown). *P < 0.05, **P < 0.01, ***P < 0.001.

In the ITT cohort (*n* = 347), there was a larger decrease in TGs after the PDP compared to controls at 18 weeks; the mean difference in changes between the groups was −0.13 mmol l^−1^ (log-transformed, 95% CI = −0.07 to −0.01, *P* = 0.016 for the interaction between diet group, time-adjusted for age and sex (unadjusted model *P* = 0.018)). The mean change from baseline after the PDP was −0.21 mmol l^−1^ (95% CI = −0.33 to −0.10); after the control diet, it was −0.07 mmol l^−1^ (95% CI = −0.15 to 0.02). Differences in LDL-C concentrations between groups were not significant: −0.04 mmol l^−1^ (95% CI = −0.16 to 0.08, *P* = 0.521 for the interaction between diet group and time, adjusted for age and sex (unadjusted model *P* = 0.504)). The mean change in LDL-C from baseline after the PDP diet was −0.01 mmol l^−1^ (95% CI = −0.08 to 0.09) and 0.04 mmol l^−1^ (95% CI = −0.05 to 0.13) for the control (Supplementary Tables 3 and 5). The changes in primary outcomes and weight and waist circumference over time are shown in Fig. 4a–d.

### Secondary outcomes

Changes in secondary outcomes at the 18-week endpoint in the ITT cohort are shown in Fig. 4c,d (weight and waist circumference). Reductions in body weight, waist circumference and glycated hemoglobin (HbA1c), and increases in diet quality (HEI score), were significantly greater after the PDP than with the control diet; differences between treatments were as follows: body weight: −2.46 kg (95% CI = −3.67 to −1.25); waist circumference: −2.35 cm (95% CI = −4.07 to −0.63); HbA1c: −0.05% (95% CI = −0.01 to −0.001); and diet quality (HEI score): 7.08 (95% CI = 5.02 to 9.15). Hip circumference, blood pressure, insulin, glucose, C-peptide, apolipoproteins A1 and B, and postprandial TGs did not differ between the groups (Supplementary Table 3).

Within-group analysis of changes in PDP versus control were as follows: weight: −2.17 kg (95% CI = −3.03 to −1.31) versus 0.30 kg (95% CI = −0.56 to 1.15); waist circumference: −2.94 cm (95% CI = −4.17 to −1.71) versus −0.59 cm (95% CI = −1.81 to 0.63); HbA1c: −0.02% (95% CI = −0.05 to 0.01) versus 0.03% (95% CI = −0.01 to 0.07); and diet quality (HEI score): 7.01 (95% CI = 5.51 to 8.51) versus −0.08 (95% CI = −1.35 to 1.50) in the PDP and control group, respectively. Changes were not different for hip circumference, blood pressure, insulin, glucose, C-peptide, apolipoproteins A1 and B, and postprandial TGs.

Changes in total protein, albumin, globulin, bilirubin, alkaline phosphatase, aspartate aminotransferase, alanine aminotransferase, C-reactive protein, tumor necrosis factor alpha and full blood count were also compared between groups at 18 weeks. None of these blood measures differed between groups, apart from mean platelet volume and absolute lymphocyte concentrations (Supplementary Table 4).

### Impact of dietary intervention on the gut microbiome

**Fig. 5:**
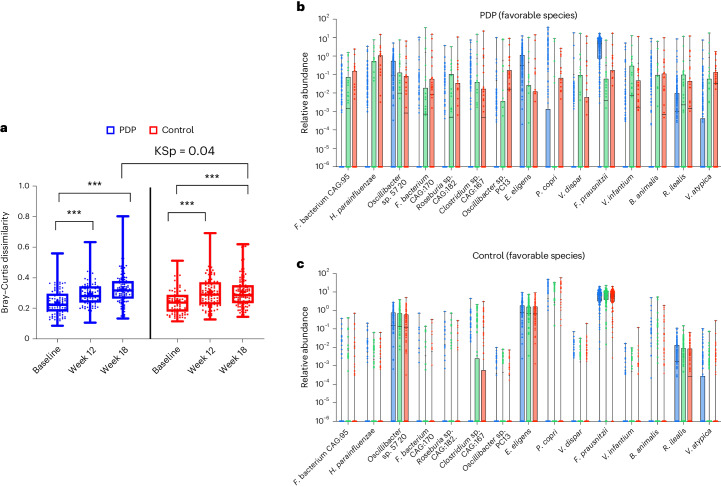
Impact of dietary intervention on the gut microbiome. **a**, Bray–Curtis dissimilarity at baseline, week 12 and week 18, for the control (red) (*n* = 118) and PDP (blue) (*n* = 112) groups. Data presented include the first quartile, median and third quartile. KSp between treatment groups (*P* = 0.04). ***P < 0.001 determined using a paired, one-sided Wilcoxon rank-sum test for within-individual change in Bray–Curtis dissimilarity. **b**,**c**, Relative abundance of favorable microbial species at baseline (blue), week 12 (green) and week 18 (red) for PDP (*n* = 112) (**b**) and control (*n* = 118) (**c**) groups (minimum to maximum shown).

The Bray–Curtis dissimilarity index (beta-diversity) was used to assess the impact of the dietary interventions on the whole microbial composition in the two groups. Bray–Curtis dissimilarities were computed in individuals with longitudinal microbiome samples available. At week 12, individuals from both control and PDP groups showed higher beta-diversity with respect to their baseline microbiome composition (Fig. 5a, Wilcoxon rank-sum test, Wp < 0.01). This suggests that regardless of the assigned intervention group, a change in diet composition with respect to the individuals’ habitual diet impacted the whole microbiome composition (Table 1 and Fig. 3). Moreover, beta-diversity comparisons in the same individuals at weeks 12 and 18 suggested an increasing trend in the PDP group but not in the control group (Fig. 5a); the median fold change of beta-diversity was greater at both weeks 12 and 18 in the PDP group than in the control group (Supplementary Table 5). Comparing beta-diversity dissimilarities across the control and PDP groups at week 18 showed a statistically significant difference (Kolmogorov–Smirnov stochasticity parameter, KSp = 0.04). In summary, the PDP intervention group showed a greater effect on the whole microbiome composition of different individuals, who were diverging more over time than the control group.

To evaluate the impact of the dietary interventions on the whole microbiome composition, we used machine learning to assess the level of associations between changes at the species level with changes in the measured health markers at the endpoint (Methods). For this analysis, we used the same machine learning framework that we developed in our previous work^13^. The results showed that variations in the relative abundance of microbiome species effectively discriminated individuals based on their changes in weight and hip circumference in the PDP intervention group, but not in the control group (area under the curve (AUC) = 0.65 and 0.59 in PDP for weight and hip circumference, respectively, and 0.49 for both in control) (Supplementary Table 6).

**Extended Data Fig. 1:**
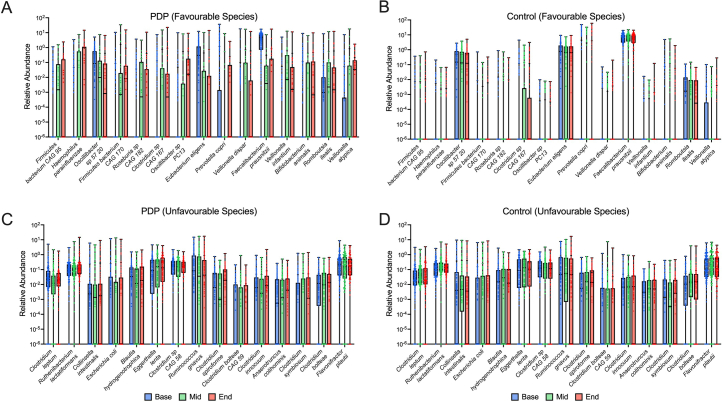
Relative abundance of microbial species. Relative abundance of the 15 favourable and unfavourable microbial species at baseline (blue), week-12 (green) and week-18 (red) for A) PDP (favourable species), B) Control (favourable species), C) PDP (unfavourable species), D) Control (unfavourable species). PDP, n = 112 and Control, n = 118 (Min to Max presented).

Finally, we examined differences in terms of relative abundances for the 30 microbial species we previously identified associated with either ‘favorable’ or ‘unfavorable’ cardiometabolic health^13^ between the two intervention groups. Notably, among the 15 favorable species, we found eight species in the PDP group showing an increase in terms of relative abundance at the endpoint (difference from baseline greater than 0); conversely, in the control group, none of the 15 favorable species showed an increased relative abundance at the endpoint (summed abundance change: 0.48 ± 9.05 versus −0.73 ± 8.63) (Mann–Whitney–Wilcoxon test, MWWp = 0.015) (Supplementary Table 7). For each of the 15 favorable species, we calculated the average fold change. We observed that 11 of the 15 favorable species showed a positive fold change in the PDP intervention group, while only four in the control group (Fig. 5b,c and Supplementary Table 7). In contrast, of the 15 previously identified unfavorable species, participants in the PDP or control intervention group did not exhibit differences in terms of changes in relative abundances (summed abundance change; 0.01 ± 3.67 versus 0.50 ± 3.43; Extended Data Fig. 1 and Supplementary Table 7). We also found that five of the 15 unfavorable species showed a decrease in fold change between the endpoint and baseline in the PDP group and four in the control group. As basic gut microbiome information, we calculated both species richness and Shannon alpha diversity measures and did not observe significant differences in the ITT group compared to the control group at week 18 (Supplementary Tables 3); because of increased taxonomic resolution availability, the value of these measures when interpreting the impact of a dietary modification or for host health is now unclear^14^.

### Safety

Adverse events were reported to the study coordinator; they were reviewed by the principal investigator and medical director. All adverse events were documented in line with institutional review board (IRB) guidelines. There were four adverse events during the study. None were classified as severe. There were no withdrawals resulting from injury. There was one withdrawal due to an undisclosed food allergy (nut allergy) that precluded further participation in the study. After consuming the test muffin, the participant experienced mild itching of the tongue and throat, nausea and an upset stomach that resolved with oral diphenhydramine. Tree nuts are not an ingredient in the test muffins but they are produced in a facility that handles tree nuts. Symptoms were graded Common Terminology Criteria for Adverse Events Toxicity grade 1 and attributed as a probable allergic reaction to the muffin. The participant had to withdraw because they were unable to complete the test meals due to their undisclosed food allergy. The other three adverse events were bruising after a blood draw (toxicity grade 1), light-headedness at the time of blood draw (toxicity grade 1) and mild bleeding from the continuous glucose monitor (CGM) (toxicity grade 2) that quickly resolved on its own.

### Energy, sleep quality, mood and hunger

As well as showing differences in clinical markers of cardiometabolic health, participants reported subjective changes in energy level, sleep quality, mood and hunger. On average, a greater proportion of PDP participants reported improvements in energy level (43% versus 11%), sleep quality (35% versus 9%), general mood (33% versus 15%) and reduced hunger levels (22% versus 14%) compared with controls (*P* < 0.01 for all) (Fig. 4e).

### Post-hoc analyses

Per-protocol analysis revealed a larger change in TGs after the PDP intervention (*n* = 108) compared to controls (*n* = 117) at 18 weeks; the mean difference in changes between groups was −0.17 mmol l^−1^ (log-transformed 95% CI = −0.07 to −0.01; *P* = 0.032 for the interaction between diet group and time, adjusted for age and sex (unadjusted model *P* = 0.032)). The mean change in TGs from baseline after the PDP intervention was −0.23 mmol l^−1^ (95% CI = −0.33 to −0.12); after the control diet, it was −0.06 mmol l^−1^ (95% CI = −0.16 to 0.05). Differences in LDL-C concentration between groups remained nonsignificant at 0.05 mmol l^−1^ (95% CI = −0.08 to 0.19; *P* = 0.430 for the interaction between diet group and time, adjusted for age and sex (unadjusted model *P* = 0.43)). For the secondary outcomes, there was a greater difference in change between diet groups in the PDP cohort. Further reductions were observed in the PDP cohort for body weight −2.51 kg (95% CI = −3.79 to −1.23) as well as increases in diet quality (HEI score = 7.32 (95% CI = 5.08 to 9.55)) (Supplementary Table 8).

We performed subgroup analysis based on dietary adherence to determine whether highly adherent participants differed across treatment. We identified adherent control participants (top 30th percentile of participants based on the HEI score, a measure of adherence to USDA dietary guidelines) and compared them to adherent PDP participants (top 30th percentile of participants based on a personalized diet quality score). Greater changes in outcomes were observed in adherent PDP versus adherent control groups for weight (−4.09 ± 4.51 versus −0.44 ± 3.27 kg, *P* = 0.002), apolipoprotein B (−7.94 ± 13.7 versus −1.14 ± 12.8 mg dl^−1^, *P* = 0.025) and total cholesterol (−0.40 ± 0.51 versus −0.13 ± 0.63 mmol l^−1^, *P* = 0.047) (Supplementary Table 9 and Fig. 4f). We also identified participants with low versus high baseline diet quality (HEI-derived top 30th percentile). When we compared changes in outcomes between these two groups, we saw no difference in any outcomes in the PDP group (high versus low baseline diet quality) or in the control group (high versus low baseline diet quality).

Given the variability in adherence, we also compared PDP participants across tertiles of adherence based on their average personalized diet quality scores throughout the intervention period. Higher adherence to the PDP was associated with greater change in several health outcomes versus low adherence to the PDP (Supplementary Table 9); greater reductions in LDL-C (−0.20 ± 0.48 versus 0.07 ± 0.56 mmol l^−1^, *P* = 0.019), waist circumference (−6.31 ± 5.35 versus −1.42 ± 5.95 cm, *P* = 0.001), diastolic blood pressure (−4.08 ± 8.56 versus 2.71 ± 9.23 mmHg), HbA1c (−0.06 ± 0.20 versus 0.02 ± 0.12%, *P* = 0.024), total cholesterol (−0.4 ± 0.51 versus −0.01 ± 0.58 mmol l^−1^, *P* = 0.002) and apolipoprotein A1 (−12.74 ± 26.2 versus 3.39 ± 15.6 mg dl^−1^, *P* = 0.001) were observed between highly adherent (*n* = 35) and low adherent (*n* = 33) participants. The proportion of participants reporting improvements in subjective hunger levels (88.6% versus 66.7%, *P* = 0.015) was also greater in highly adherent PDP participants versus low adherent participants. Highly adherent PDP participants had an average weight loss of 4.7% versus 2.4% compared to low adherent participants (*P* > 0.05). When PDP participants were stratified based on their baseline LDL-C concentration (unhealthy, 3.4 mmol l^−1^ or greater; healthy, less than 3.4 mmol l^−1^), those with unhealthy baseline levels showed decreasing trends in LDL-C across both adherence groups (low, −0.07 ± 0.18; high, −0.07 ± 0.13 mmol l^−1^, *P* = 0.866), whereas in those with healthy baseline levels, only highly adherent participants had a significantly greater mean decrease (high, −0.03 ± 0.15 mmol l^−1^, *P* = 0.008).

## Discussion

In this randomized, controlled trial of an 18-week dietary intervention in adults, when compared with US standard care dietary advice, a PDP intervention resulted in greater improvements in diet quality, which also resulted in greater reductions in TG concentration, weight, waist circumference and HbA1c, but not LDL-C. It also favorably shifted the gut microbiome composition, as well as subjective feelings of hunger, energy and mood, demonstrating another potential benefit of a PDP in improving overall health and well-being. Overall, these findings suggest that a dietary program focused on personalized advice is more effective in reducing central adiposity and TG concentrations than standard dietary advice in generally healthy individuals.

The PDP led to greater reductions in TG levels versus a control diet. While TGs improved, LDL-C did not differ between the groups at 18 weeks, similar to previous personalized nutrition evidence^11^. However, LDL-C was reduced in highly adherent PDP participants. When participants were further stratified based on their baseline LDL-C, those with unhealthy baseline levels (3.4 mmol l^−1^ or greater) showed decreasing trends in LDL-C across all adherence groups. While in participants with healthy baseline levels (less than 3.4 mmol l^−1^), only highly adherent participants had a significantly greater mean decrease. These findings suggest that high adherence to a PDP may reduce LDL-C in most participants; clearer effects may have been observed if conducted in participants with hyperlipidemia. These findings are not surprising as recent evidence showed that TG levels are more sensitive to nutritional intervention; additionally, LDL-C levels may not change with weight loss induced by dietary modification^4,15^. Livingstone et al.^16^ demonstrated the efficacy of personalized nutrition in modifying dietary intakes depending on the clustering of adherence to dietary recommendations. Individuals with the poorest diets benefited the most from a personalized nutrition intervention. Conversely, in this study, we did not observe greater health improvements in participants with lower baseline diet quality. Additionally, we saw greater improvements in adherent PDP versus control participants, which may further support the effect of a personalized nutrition-based treatment independent of adherence and baseline diet.

Our findings support the application of this PDP over generalized guidance for the purpose of improving body weight and waist circumference, despite favorable dietary changes including increased fiber intake in the control group. Previous personalized approaches have not reported greater improvements in body weight on a personalized diet versus control. For example, the Food4Me European study of personalized nutrition^10^ showed improved dietary behaviors (that is, HEI), but no significant differences in body weight at 6 months when compared with a nonpersonalized diet group. In addition, Ben-Yacov et al.^11^ showed no differences in body weight between a postprandial targeting diet and a Mediterranean diet at 6 months, although both groups experienced weight loss. The Personal Diet Study^17^ leveraged the predictive machine learning algorithm developed by Zeevi et al.^18^; when compared to a standardized low-fat diet, they did not report differences for weight. The PREVENTOMICS study demonstrated no additional benefit of personalizing dietary plans based on metabolic clusters, over a control group, on the change in fat mass or body weight^19^. In our multilevel approach to personalization, the weight loss observed was moderate and below proposed clinically meaningful thresholds (5%)^20^; however, moderate weight loss of this magnitude has been reported to improve health outcomes^21^. Additionally, evidence shows that the rate of weight loss observed, despite no calorie restriction advice, is likely to be sustained and meaningfully contribute to long-term health^22^. Furthermore, in highly adherent PDP participants weight loss was greater and closer to clinically meaningful levels (4.7%). Our waist circumference reduction was consistent with a magnitude associated with a reduction in cardiometabolic risk factors^23–27^. The small but statistically significant positive effect on body weight and waist circumference may reflect the impact of reducing multiple postprandial responses personalized to an individual and the greater satiating capacity reported by participants or lower energy density of the diet^28^.

The gut microbiome has a central role in human health and disease, specifically cardiometabolic health^29,30^. A bidirectional relationship exists between the microbiome and diet, whereby the gut microbiome affects host metabolism and response to foods^31^, and diet affects gut microbiome composition and functionality, which in turn exerts downstream effects on human health^32^. We demonstrated that the PDP diet had a greater and more sustained impact in shaping the whole gut microbiome composition. This change in microbiome composition was consistent with the greater change in diet quality (HEI) in the PDP group compared with controls. More specifically, we showed that the PDP diet induced favorable changes in species previously associated with favorable cardiometabolic health and diet^13^ compared to controls. At the same time, it did not impact the contribution in the microbiome of previously reported unfavorable species that were instead increased in the control group. In agreement with previous evidence^33^, we showed that changes in microbiome composition of participants after the PDP were more predictive of weight loss and hip circumference than controls. We did not see clear differences in changes of measured gut richness; however, with the increased taxonomic resolution available from MetaPhlAn 3.0, previous research questioned whether this is a valid measure of host health^14,34^.

One criticism of personalized advice is that the resulting variance in nutrient intake is low and the advice pushes all individuals toward the same dietary pattern and similar changes in nutrient intake. For example, the study by Ben-Yacov et al.^11^, in adults with prediabetes, demonstrated that a personalized diet resulted in most participants adopting lower carbohydrate and higher protein and fat intakes compared with those randomized to a Mediterranean diet. In the PDP group, while we observed small average decreases in carbohydrate intake and increases in healthy fat (polyunsaturated fats) intake, we saw a large variation in nutrient intake and individual foods, which is not captured by the mean cohort intake. Because this PDP was developed using multiple inputs, including postprandial fat and glucose as well as the microbiome, it does not push all participants toward a low carbohydrate diet. However, we also acknowledge that the composition of foods is more nuanced than their nutrient composition, such that the matrix^35,36^ and processing level of foods^37^ can have major effects on health.

This study tested the first version of a prediction algorithm, developed in 2022, which could be further advanced by personalizing the generalized lifestyle and dietary habit advice that complement personalized diet scores. Evidence showed that health is affected by interlinked factors, including dietary intake, underlying physiological status and the interaction between diet and behaviors such as lifestyle, meal context, time of day, exercise and sleep. For example, we showed that poor sleep efficiency, later bedtimes (midpoint) and deviation from habitual sleep patterns are associated with poorer postprandial glycemic control^38^. Time of day or eating window duration also has implications for dietary responses^39–41^, such that eating later induces nocturnal glucose intolerance and reduces fatty acid oxidation and mobilization, independently of sleep^39^. Food and meal order, including consuming carbohydrates before protein and vegetables in a meal, contributes to elevated glycemic variability^42^. The protective effects of physical activity on responses are well established^43^, with evening exercise eliciting lower lipemic responses to high-sugar breakfasts the next day in postmenopausal females^44^. All of these factors present modifiable behavioral strategies and show the interaction between diet and behaviors. This suggests that a future PDP based on a more representative cohort (more than 100,000 participants) with personalization on lifestyle (for example, physical activity, sleep) and dietary behaviors might deliver even greater improvements in outcomes.

The strengths of this study include it being conducted in generally healthy middle-aged and older males and females broadly representative of the US population, not young healthy individuals. Although the average BMI for this cohort was 34 kg m^−^^2^, central obesity (using sex and ethnicity-specific waist circumference cutoffs) was representative of the average US population and participants were not receiving lipid-lowering or blood glucose-lowering medications (that is, statins and antidiabetic medications), which is a strength because evidence is lacking for prevention in populations without diabetes. The study was run remotely and participants were free-living, so the result is more reflective of real-world settings than a traditional clinical trial design approach.

Limitations include that we could not accurately capture changes in physical activity status. Furthermore, although reflective of how the advice is delivered in real life, the USDA recommended diet was delivered via leaflet and video, and was intentionally not matched for contact or intensity with the PDP group. These differences between treatments should be considered when interpreting the results. Future studies would benefit from assessing the impact of a personalized program versus personalized food scores. However, the control group slightly improved fiber intake and reduced fat consumption, and were aware that they were in a trial, which we know influences behavior. Although the PDP was well received by participants, and study participants were recruited from the general population, a larger study is required to capture more diverse ethnicities and for better gender representation. This study is also not applicable to children or old adults.

In conclusion, a personalized nutrition program that addresses metabolic heterogeneity is effective in improving cardiometabolic health in generally healthy individuals. The results demonstrates that a PDP underpinned by multiple biological inputs (glucose, TGs, microbiome, cardiovascular disease risk and health history) and overlaid with generalized dietary and lifestyle advice improves TG concentrations substantially more than a standard USDA diet and may contribute to the overall reduction in risk of cardiometabolic diseases.

## Methods

### Study design

The ZOE METHOD study was an 18-week parallel-design, randomized controlled trial. The trial was registered on ClinicalTrials.gov (ClinicalTrials.gov registration: NCT05273268) and listed as the ZOE METHOD Study: Comparing Personalized versus Generalized Nutrition Guidelines. The remote trial carried out in the US compared standard care dietary advice (control) versus a PDP in a cohort generally representative of the US adult population. Standard care dietary advice (United States Dietary Guidelines for Americans, 2020–2025) was delivered in the form of an USDA dietary recommendations digital leaflet, a short video lesson, access to online resources and regular check-ins. The PDP provided dietary advice using the ZOE 2022 algorithm, incorporating food characteristics, individuals’ glucose control and postprandial TG concentrations^3^, individuals’ microbiomes^13^, atherosclerotic cardiovascular disease risk and health history, to produce personalized food scores delivered during an 18-week program alongside more generalized nutrition and lifestyle education through a remote mobile phone application (the ZOE app). Ethical approval for the trial was obtained through the Advarra IRB (IRB no. 00000971; protocol no. 00044316). All participants provided written informed consent and the study was carried out in accordance with good clinical practice and the Declaration of Helsinki (2013). Outcome measurements were made at baseline and after randomization to their respective treatments.

### Participant selection and randomization

Males and females reflective of the average US adult population (aged 40–70 years; waist circumference greater than ethnicity-specific and sex-specific 25th percentile values; fruit and vegetable intake below 450 g per day (to capture 75% of the population)) living in the US were recruited (1 March 2022 to 10 August 2022) by electronic advertisement (e-mail to the Stanford Nutrition Studies Research Cohort, the Empowered Gut newsletter and the ZOE Ltd mailing lists). Both sexes were eligible for recruitment and sex was determined using self-reported questionnaires with the following question: ‘What sex were you assigned at birth?’ Through the recruitment channels (e-mail and website), participants were invited to complete an online screening questionnaire and then invited to attend a primary baseline clinical visit (described in detail below) where all eligibility criteria were assessed. After this two-step screening process, participant eligibility was confirmed and a minimization-randomization program (MinimPy v.0.3, Python Package Index; pypi.org/project/MinimPy/) was used for treatment allocation. Participants were randomly and equally allocated to one of the two treatments based on the following minimization factors: (1) sex, male or female; (2) waist circumference, above or below their ethnicity-specific median; and (3) fruit and vegetable intake, above or below the median US adult intake of 234 g per day. Trained study coordinators enrolled, assigned and informed participants about their allocation to treatment via e-mail. Participants were informed of all study procedures before providing electronic consent. Participants were excluded from the study if any of the following criteria applied: had taken part in the ZOE product or any PREDICT study beforehand; were unable to read and write in English, as the ZOE app is only available in English; did not complete the first Quest visit successfully; had an iOS/Android device not compatible with the app; used medications affecting lipids (lipid-lowering drugs, for example, statins; antidiabetic medications, for example, metformin and insulin), and supplements including fish oil (unless willing to safely come off these for 4 weeks before the start of the study, and for the duration of study); had ongoing inflammatory disease, for example, rheumatoid arthritis, systemic lupus erythematosus, polymyalgia and other connective tissue diseases; had cancer in the last 3 years, excluding skin cancer; had chronic gastrointestinal disorders, including inflammatory bowel disease or celiac disease (gluten allergy), but not including irritable bowel syndrome; were taking the following daily medications: immunosuppressants, corticosteroids or antibiotics in the last 3 months, not including inhalers; were users of prescription proton pump inhibitors, such as omeprazole and pantoprazol, unless they were able to stop 2 weeks before the start of the study and remained off them for the entire duration of the study (provided their treating physician deemed it safe for them to do so); were currently suffering from acute clinically diagnosed depression or anxiety disorder; had a heart attack (myocardial infarction) or stroke in the last 6 months; were pregnant or planning pregnancy in next 12 months, or were breastfeeding; were vegan, had an eating disorder or were unwilling to take foods that were part of the study; had an allergy to adhesives, which would prevent proper attachment of the CGM.

### Interventions and procedures

The study design is summarized in Fig. 1.

#### Primary baseline testing (week −1)

##### Baseline clinical visit

Participants attended a baseline clinical visit at the Quest Diagnostic Patient Service Center, where baseline measures were assessed, including a fasted venous blood draw, and anthropometric measurement of height, body weight, hip circumference, waist circumference and blood pressure. Participants who did not attend a clinic visit within 1 week of their visit date were withdrawn from the study.

##### Health questionnaire

Participants remotely completed two questionnaires administered through an online survey before randomization. These questionnaires included (1) a primary questionnaire capturing baseline health status and medical health history and (2) a secondary questionnaire capturing information on anthropometrics, sleep, energy level, mood, hunger, skin, female health (menopause) and current medication use.

##### Participant survey

A survey where participants confirmed completion of the primary baseline study tasks was administered at the end of week −1 to assess participant compliance.

##### Stool sample collection

Stool samples for microbiome analysis (required for the algorithm predictions) were collected by participants at home using the DNA/RNA SheildTM Fecal Collection Tube (Zymo Research) containing buffer (catalog no. R1101, Zymo Research). Once collected, the sample was stored at room temperature before being shipped to the analyzing laboratory inside a prepaid return kit.

#### Secondary baseline testing (week 0)

##### Baseline measures

After allocation to treatment, both PDP and control groups completed a secondary set of baseline measurements, including fasted venous blood tests, questionnaires and stool collection as described in the primary baseline testing section. Approximately 1 week after their primary clinical visit, participants completed a secondary visit to the Quest center. Non-completion of this second visit within the required time period resulted in participant withdrawal from the study. In addition to this, participants completed an FFQ. The PREDICT FFQ, which captured information on 264 foods, food groups and beverages over the previous month was administered via an online survey^3^. For the control group, links to the FFQ were provided via e-mail. For the PDP group, links to the FFQ were provided via e-mail or via the ZOE app.

##### ZOE test kit

PDP participants were additionally asked to complete the ZOE test kit. This included (1) a CGM, (2) standardized test meals (three muffins) and (3) a DBS. Participants applied and wore a CGM (Freestyle Libre 2, Abbott) on their upper arm for up to 14 days. Two days after CGM application, participants completed 2 days of standardized meal intervention. Meals consisted of muffins with mixed macronutrient composition and were consumed for breakfast and lunch (day 1, as a sequential mixed meal intervention) and for breakfast only (day 2). Breakfast meals were consumed after an overnight fast of at least 8 h. Participants were asked to consume the entire portion of the meal provided within 15 min. The consumption of their meal was scanned in the app using the unique barcode labeled on each meal. The time participants started and completed eating their meal was recorded. They were asked to report any deviations from this protocol to study staff.

After the sequential test meal, a finger-prick DBS test was completed (6 h after breakfast to measure postprandial responses). Blood test cards were stored at room temperature until shipping to the analyzing laboratory via a prepaid return mailing kit. Finally, after completion of their test meals, participants were asked to log their habitual diet through the ZOE app. This app provided the functionality of a weighed food diary as well as a log of all the study tasks required of the participant during the ZOE test kit phase.

##### Dietary advice

Participants in the control group were e-mailed a PDF file containing a digital leaflet from the USDA Dietary Guidelines for Americans (2020–2025) accompanied by a video verbalizing the dietary advice, in accordance with a typical general consultation. In addition, participants were provided with online resources. Study coaches were available by e-mail to answer questions and provide support. The USDA guidelines recommend daily or weekly amounts from different food groups to maintain a healthy lifestyle. Participants were advised to follow this dietary advice for the study duration (weeks 2–18). Each week they received an e-mail from a study nutrition coach to check in.

PDP participants received generalized nutrition and lifestyle advice through the ZOE app, which they followed for 4 weeks (weeks 2–6), while personalized results were being generated via the ZOE 2022 algorithm (see Fig. 1 for more details). Generalized advice was presented via the app in the form of interactive ‘lessons’ as part of a program of learning. The lessons covered basic nutritional and dietary health concepts, including dietary diversification, increasing plant food consumption, increasing fiber intake, replacing refined carbohydrates with wholegrains and consumption of fermented foods.

At week 6, PDP participants received a personalized ‘Insights’ study report, including a personalized blood sugar score, blood fat score, gut diversity score, gut microbiome score and presence or absence of several microbial species^2^. These reports also included results from the ZOE 2022 algorithm, specifically information about person-specific food scores.

The interventions were not matched for contact or intensity to test the efficacy of the PDP, which involves personalized diet scores overlaid with generalized dietary and lifestyle advice delivered as a set of program lessons.

##### Personalized food quality scores

A personalized ZOE food quality score was computed using the ZOE 2022 algorithm for each food item consumed by the PDP participants. Food quality scores were based on both the macronutrients of a food item and further food metadata, including glycemic load, fat quality, level of processing and food group (for example meat, fruit, vegetables and fermented foods). They were personalized to an individual’s glucose control, postprandial TG concentration, atherosclerotic cardiovascular disease risk, health history and microbiome composition (abundance of specific health-promoting and health-reducing microbial taxa and the associations of these taxa with food items). The ZOE 2022 algorithm was trained using expert input on appropriate food quality scores for different individual phenotypes for a small number of foundational foods, and was used to predict personalized food quality scores for all individual phenotypes and all food items, which were then further personalized for detailed microbiome composition.

The food quality scores ranged from 0 to 100, with higher values indicating more healthful meals. Based on this food quality score, personalized recommendations could be made, that is, consume foods with a quality score of 0–24 once in a while, enjoy in moderation foods with a score of 25–49, enjoy foods regularly with a score of 50–74 and enjoy foods freely with a score of 75–100. A participant’s personalized meal scores throughout the day were combined by further algorithms to generate personalized day scores also ranging from 0 to 100. Throughout the study, participants were instructed to consume a diet (and record it in the app) reaching a certain day score threshold, which increased throughout the study duration, to the best of their ability. These day scores were accessible to participants, aiming to motivate them and convey to them their compliance to their dietary advice. The diet did not involve calorie restriction or calorie counting.

From week 6, PDP participants received personalized food scores and meal recommendations within the ZOE app. PDP participants were asked to attend a single phone or video call with a study staff member to discuss their results and to make these results immediately accessible to and actionable by the participant. Following this, a set of program lessons was administered in the app for 12 weeks (termed the ‘action plan’) during which participants were taught how to engage with and adhere to their personalized plan. Contact with study coaches was available via the app.

#### Week 12 measures

PDP and control groups completed a set of measures at week 12, including fasted venous blood tests (Quest visit), questionnaires, stool collection and FFQ as described in the primary and secondary baseline testing sections above.

#### Endpoint measures (week 18)

Endpoint data collection was completed in the 19th week of the study, at which point both groups had been allocated to their respective treatments for 18 weeks. PDP and control groups completed a set of endpoint measures, including fasted venous blood tests (Quest visit), questionnaires, stool collection and FFQ as described in the primary and secondary baseline testing sections above.

PDP participants were provided a second ZOE test kit to retest their nutritional responses, including application of a second CGM, consumption of the standardized meal intervention and completion of DBS.

#### Additional follow-ups

PDP participants were followed up at 8 and 12 months with a clinical visit, including fasted venous blood tests, questionnaires, stool collection and FFQ as described in the primary and secondary baseline testing sections above. Control participants were given the option to join a nested cross-over arm on completion of the 18-week endpoint measures. These participants completed the PDP arm protocol and completed the 6-, 12- and 18-week measures. Alternatively, control participants were offered the ZOE nutrition commercial product.

Participants were recruited from March 2022 to August 2022. The core intervention period took place from April 2022 to February 2023, and follow-ups were completed by September 2023.

### Adherence

As part of the study design, participants in both arms were asked to self-report adherence (scale 0–10) to the dietary advice given by the questionnaire administered every 6 weeks (week 7, week 12 and week 18 for the control group; week 12 and week 18 for the PDP group) during the study period. As part of the PDP only, participants were asked to record their dietary intake in real time on a minimum of four consecutive days (including one weekend day and 1,200 kcal or more per day) per month using a designated smartphone app (ZOE app). Each food item was recorded along with weight or portion units by selecting the food from a database (the USDA compositional database and a commercial database) containing approximately 900,000 items. Adherence to the PDP was evaluated through logging metrics and self-recorded dietary intake in the logging app.

### Outcomes

Specified primary outcomes were serum TG concentration and direct LDL-C concentration. The primary outcome was the 18-week change from baseline. Therefore, secondary outcomes were changes in weight, waist circumference, hip circumference, systolic blood pressure and diastolic blood pressure, blood HbA1c, serum insulin, serum glucose, serum C-peptide, serum apolipoprotein A1, serum apolipoprotein B, fecal gut microbiome (species richness, Shannon diversity and Bray–Curtis dissimilarity), postprandial blood TG concentration, habitual diet quality (HEI) and self-reported energy level. Other outcomes included self-reported mood, hunger, total protein, albumin, globulin, bilirubin, alkaline phosphatase, aspartate aminotransferase, alanine aminotransferase, C-reactive protein, tumor necrosis factor alpha and full blood count.

### DBS collection and processing

Postprandial TG (mmol l^−1^), high-density lipoprotein cholesterol (mmol l^−^^1^) and cholesterol (mmol l^−1^) were quantified from finger-prick DBS (Clinical Reference Laboratory) tests completed by PDP participants in weeks 0 and 18 of the study (during completion of the ZOE test kit). DBS tests were completed 360 min after consuming the breakfast test meal. After washing their hands, participants pricked a finger with a sterile lancet and placed 3–4 drops of blood on their test card. Study staff assessed test validity using a photo and time point of testing logged by the participant in the app. Test cards not meeting the quality protocol (multiple small spots or inadequate coverage) were not included in the analysis. Participants were encouraged to complete the sequential test meal and DBS test again when either of these was inadequately completed. Each test card was stored in a foil pouch with a desiccant packet once completed and mailed to the analyzing laboratory in a prepaid kit within 24 h of completion.

Analysis was done at the Clinical Reference Laboratory. Advance Dx100 Technology DBS cards were analyzed for lipemic metabolites by the Clinical Reference Laboratory. Portions of test cards were taken from the sample, from which the dried blood was extracted and analyzed using standard quantification methods.

### Fasted venous blood collection and processing

Fasted venous blood draws were performed at Quest Diagnostic Patient Service Centers and processed by Quest Diagnostics; 500 μl of venous blood was collected in serum separator tubes (SSTs). Then, 250 μl of venous blood was collected in EDTA tubes. SSTs and EDTA tubes were left at room temperature for 30 min (or up to 1 h) and centrifuged at 1,600*g* for 15 min at 4 °C. Direct LDL-C, TG, glucose, insulin, C-peptide, apolipoprotein A1 and apolipoprotein B were quantified in serum (SST), and HbA1c was quantified in whole blood (EDTA). The full list of clinical blood chemistry measures quantified in this study are shown in Supplementary Table 10.

### Continuous glucose monitoring

Interstitial glucose was measured every minute and aggregated into 15-min readings, using the Freestyle Libre 14-day CGM (Abbott Diabetes Care). Participants randomized to the PDP group were instructed to apply the CGM two days before starting their standardized meal intervention, to the upper, nondominant arm and to cover the monitor with an adhesive patch (Sourceful) for improved durability. CGMs were worn for up to 14 days and participants were unblinded to the results. Given that the CGM device requires time to calibrate once applied, CGM data collected 12 h and onwards after activating the device was used for the analysis.

### Fecal sampling and microbiome testing

#### DNA extraction and sequencing

On receipt in the laboratory, samples were homogenized, aliquoted and stored at −80 °C in QIAGEN PowerBeads 1.5-ml tubes and used to extract bacterial DNA. All 815 stool samples were processed and analyzed using a Shotgun Metagenomic Sequencing Service (Zymo Research). The DNA was first isolated using the ZymoBIOMICS 96 MagBead DNA Kit (Zymo Research). Then, the sequencing libraries were prepared using the Illumina DNA Library Prep Kit with up to 500 ng DNA input according to the manufacturer’s protocol, using unique dual-index 10-bp barcodes with Nextera adapters (Illumina). The libraries were pooled in equal abundance and the final pools were quantified using quantitative PCR and a TapeStation (Agilent Technologies). The final libraries were sequenced using the NovaSeq 6000 platform (Illumina) according to the manufacturer’s protocols, generating 150-bp paired-end reads. The NovaSeq control software NCS v.1.5 was used. Image analysis, base calling and quality checking were performed with the Illumina data analysis pipeline RTA3.3.5 and bcl2fastq v.2.20.

#### Metagenome quality control and preprocessing

All sequenced metagenomes were preprocessed using the pipeline implemented in github.com/SegataLab/preprocessing. Briefly, the pipeline consisted of three steps: the first step involved read-level quality control and removed low-quality reads (*Q* < 20), too-short reads (less than 75-bp long) and reads with more than two ambiguous nucleotides. The second step screened for contaminant DNA using Bowtie 2 (ref. ^45^) with the ‘--sensitive-local’ parameter, allowing confident removal of the phi X 174 Illumina spike-in and human-associated reads (hg19 reference human genome release). The last step consisted of splitting and sorting the cleaned reads to create standard forward, reverse and unpaired read output files for each metagenome (average: 35 ± 13 million reads per sample).

#### Microbiome taxonomic profiling

Species-level profiling of the 815 samples was performed with both MetaPhlAn 3.0 (ref. ^34^) and MetaPhlAn 4.0 (ref. ^46^). Default parameters were used for both versions of MetaPhlAn, while specific databases to each version were used, mpa_v30_CHOCOPhlAn_201901 and mpa_vJan21_CHOCOPhlAnSGB_202103 for version 3 and 4, respectively. MetaPhlAn 3.0 taxonomic profiles were used to assess the presence and contribution of the previously identified 15 positively associated and 15 negatively associated species with dietary and cardiometabolic health markers^2^. MetaPhlAn 4.0 taxonomic profiles were analyzed to compare microbial compositions between participants and to determine alpha diversity indices, the number of detected species (observed richness). Microbiome taxonomic profiles were also analyzed to compare between-microbiome-sample dissimilarity (beta-diversity) using the Bray–Curtis dissimilarity measure.

#### Machine learning

We used the same machine learning framework developed by Asnicar et al.^13^ to assess the link of the microbiome compositions with the different dietary and metabolomic outcomes. Briefly, the machine learning framework is based on the random forest classification and regression algorithms and a 100-fold cross-validation approach with a 80/20 random splitting of the dataset. As training data, we used the differences in relative abundance between the 18-week and baseline time points of only microbial species. The classification task was evaluated using the area under the receiver operating characteristic curve, while the regression was evaluated by correlating the predicted values with the target values using the Spearman correlation coefficient.

### Diet information

Participants completed the PREDICT FFQ online, at three separate time points throughout the study (0 weeks, 12 weeks and 18 weeks) to capture habitual dietary intake over the preceding month. The FFQ included 264 food and beverage items for which the participant selected frequency of consumption over the last month. Each survey item was accompanied by an USDA standard portion size, a textual description of the portion and a photograph of the item displayed on standard size tableware. The nutritional composition of each item was allocated according to the matching, or equivalent, item composition in the USDA database^47^; US nutrient intake, including macronutrient and micronutrient data, was calculated per participant. Submitted FFQs were excluded if more than ten food items were left unanswered, or if the total energy intake estimate derived from the FFQ as a ratio of the individual’s estimated basal metabolic rate (determined using the Schofield et al.’s equation^48^) was more than 2 s.d. outside the mean of this ratio (less than 0.15 or more than 2.04). Food energy density was calculated as the ratio between food energy (kcal) and food weight (g), excluding caloric (such as milk and juices) and noncaloric beverages^28^.

### Safety

Adverse events were reported to the study coordinator, and were reviewed by the principal investigator and medical director. All adverse events were documented in line with IRB guidelines. The dietary intervention was anticipated to cause none to minimal discomfort. Some people may be affected by a small change in diet, for example, they may experience gas or bloating after eating the standardized test meals.

### Sample size calculations

The study was powered on a sample size of 150 participants per group (*n* = 300) at 90% power and *P* < 0.05, to detect a 0.21 mmol l^−1^ between-group difference in TG (endpoint change from baseline). An s.d. of 0.55 mmol l^−1^ was assumed on the basis of earlier data^49^. The same sample size was also powered to detect a 0.30 mmol l^−1^ change in LDL-C at 90% power and *P* < 0.05, assuming an s.d. of 0.8 mmol l^−1^ (ref. ^49^). Given two primary outcomes, statistical significance was defined by *P* < 0.025.

### Statistical analysis

Analyses were carried out using v.4.0.2 of R and Python v.3.9.7. Pandas v.1.1.3, NumPy v.1.23.5 and SciPy v.1.11.1 were used to manage and preprocess data. Analyses of 18-week changes in primary and secondary outcomes were conducted based on an ITT (*n* = 347). We conducted a per-protocol analysis using the data collected from participants who returned to their endpoint visit as prespecified in the protocol (18 ± 2 weeks) (*n* = 225; 65% of the ITT cohort). An average of the two clinical blood chemistry baseline samples was used as the baseline measure for each participant. The primary outcome was the 18-week changes from baseline. The comparison between treatments in continuous variables over time was performed using repeated measures analysis ensuring that all ITT participants randomized with baseline information were included in the analysis and analyzed according to the original treatment assignment. The model evaluates the interaction between time (within-subject factor) and diet treatment (between-subject factor) with diet treatment, time, age and sex included as fixed effects along with a random effect for participants. The intervention effect was the coefficient for the interaction term in the model and the associated 95% CIs. The simple main effects of differences between the two diet groups were also assessed. For outcomes that were not normally distributed, outcomes were log_10_-transformed and tested for normality using the Shapiro–Wilk test. Given two primary outcomes, statistical significance was defined by *P* < 0.025. The between-group analysis was performed by a blinded researcher. Group allocation was concealed by labeling the groups with nonidentifying terms.

We assessed gut microbiome composition using species-level taxonomic profiles of participants with longitudinal sampling available. The ITT cohort was restricted to 118 and 112 individuals for the control and PDP groups, respectively. For each individual, we calculated the within beta-diversity using the Bray–Curtis dissimilarity index between the longitudinal samples available. For the baselines (week −1 or week 0), when two samples were available for the same individual, we considered the one with the highest number of preprocessed reads. As reference beta-diversity variability for comparison with the week 12 and week 18 samples, we considered the values calculated in each individual with the two baseline samples available (both week −1 and week 0). Bray–Curtis dissimilarities of the longitudinal samples of the same individuals between control and PDP groups were tested using a paired, one-sided Wilcoxon rank-sum test, while across-intervention groups were tested using a Kolmogorov–Smirnov stochasticity parameter (KSp). As we previously identified microbial bacterial species associated with favorable and unfavorable cardiometabolic risk markers^13^, we tested differences between the two intervention groups. We tested statistically significant differences in terms of relative abundance values for favorable and unfavorable species between groups using a Mann–Whitney–Wilcoxon test (MWWp) and reported the magnitude and direction of change using a log_2_ fold change.

We performed a subgroup analysis based on dietary adherence to determine whether highly adherent participants differed across treatments. We identified adherent control participants (top 30% of participants based on the HEI score, a measure of adherence to USDA dietary guidelines) and compared them to adherent PDP participants (top 30% of participants based on a personalized diet quality score). Adherence to the ZOE program was classified based on a mean personalized diet score throughout the study duration. A minimum of 4 days of logged diet data meeting sex-specific caloric cutoffs (females, 500–5,000 kcal or more per day; males, 500–8,000 kcal or more per day) was required per month to ensure high quality and quantity logging. Low adherent participants were classified as the bottom 30th percentile of participants (mean personalized day score of 58 or lower); highly adherent participants were the top 30th percentile (mean personalized day scores of 67 or greater); moderately adherent participants fell in the middle (mean personalized day scores of 59–66). We also conducted a within-PDP analysis to investigate whether participants with good adherence (top 30%) to the PDP personalized dietary advice showed greater improvements in health outcomes compared to those with poor adherence (bottom 30%). Sex-based analysis was not performed because of small sample sizes. Excel v.16.82 and Microsoft Office were used for data and table formatting.

### Reporting summary

Further information on research design is available in the Nature Portfolio Reporting Summary linked to this article.

## Online content

Any methods, additional references, Nature Portfolio reporting summaries, source data, extended data, supplementary information, acknowledgements, peer review information; details of author contributions and competing interests; and statements of data and code availability are available at 10.1038/s41591-024-02951-6.

### Supplementary information

Supplementary Information Supplementary Tables 1–10.

Reporting Summary

## Data Availability

The study data can be released to bona fide researchers submitting a research proposal approved by a subpanel of our scientific advisory board. We have meetings once per month with independent members to assess proposals. The data will be anonymized and conform to UK General Data Protection Regulation standards. Access request proposals should be sent to data.papers@joinzoe.com. The microbiome data will be uploaded onto the EBI website (www.ebi.ac.uk/).

## Code availability

The scripts for the statistical analysis are freely available upon request to ZOE Ltd. Application is via data.papers@joinzoe.com. Code will be made available within 2 months of the request.
