# Supplementary material for: Effects of a personalized nutrition program on cardiometabolic health: a randomized controlled trial
Source: Nat Med. 2024 May 8;30(7):1888–97. doi: 10.1038/s41591-024-02951-6 (PMC11271409; doi:10.1038/s41591-024-02951-6)
Supplement: Supplementary file 1 — Supplementary Tables 1–10. [file 41591_2024_2951_MOESM1_ESM.pdf]

nature medicine

Article

https://doi.org/10.1038/s41591-024-02951-6

Effects of a personalized nutrition program on cardiometabolic health: a randomized controlled trial

In the format provided by the authors and unedited

Table of Contents

Supplementary Table 1	Description of dietary intake in the METHOD participants by randomized treatment group (ITT cohort N=349)
Supplementary Table 2	Average personalised diet scores in the PDP group over the study months and when stratified across adherence groups.
Supplementary Table 3	Description of primary and secondary outcomes in the METHOD participants by randomized treatment group (ITT, N=349)
Supplementary Table 4	Description of additional outcomes in the METHOD participants by randomized treatment group (ITT, N=349)
Supplementary Table 5	Median fold change in the bray-Curtis dissimilarity at week-18 versus baseline.
Supplementary Table 6	Machine learning approach assessing the relationship between change in microbiome composition and change in cardiometabolic health markers across treatment groups
Supplementary Table 7	Relative abundances of 30 microbial species and change in the PDP and control groups.
Supplementary Table 8	Description of cardiometabolic health measures and changes after the personalised diet and control diet (Per protocol analysis, N=225)
Supplementary Table 9	Stratification by adherence across treatments
Supplementary Table 10	Outcome measures assessed in the ZOE METHOD study

Table 1. Description of dietary intake by visit in the ZOE METHOD participants by randomized treatment group (ITT cohort N=349)

	PDP			Control			Mean Between-Group Difference (95% CI)a	Difference in change between groups
	N	Mean	SD	N	Mean	SD		
**Total Energy Intake, kcal**								
Baseline	135	1873	627	143	1803	571	84.6 (-58.19, 227)	
Week-18	112	1648	591	121	1713	613	-77.6 (-229, 74.0)	162 (22.0, 302)
**Carbohydrates, g**								
Baseline	135	193	82	143	182	76	12.6 (-5.37, 30.6)	
Week-18	112	159	64	121	178	79	-20.9 (-40.1, -1.66)	33.5 (14.4, 52.5)
**Carbohydrates, kcal**								
baseline	135	41	9.1	143	39.9	8.9	1.27 (-0.73, 3.28)	
Week-18	112	38.5	7.6	121	41	8.2	-2.65 (-4.76, -0.53)	3.92 (2.06, 5.77)
**Fat, g**								
Baseline	135	91.3	34.4	143	89.5	32	2.45 (-5.45, 10.35)	
Week-18	112	85	33.9	121	83.2	33.2	1.42 (-6.90, 9.74)	1.03 (-6.02, 8.06)
**Fat, % energy**								
Baseline	135	43.7	7.6	143	44.5	6.9	-0.88 (-2.51, 0.74)	
Week-18	112	46.1	6.1	121	43.7	6.7	2.57 (0.84, 4.30)	-3.45 (-5.09, -1.81)
**Protein, g**								
Baseline	135	75.1	25.7	143	72.5	22	2.92 (-2.78, 8.62)	
Week-18	112	66.8	25.6	121	67.9	22.8	-1.95 (-8.01, 4.10)	4.88 (-0.79, 10.51)
**Protein, % energy**								
Baseline	135	16.3	3.2	143	16.5	3.4	-0.24 (-0.98, 0.51)	
Week-18	112	16.3	2.8	121	16.2	2.9	0.05 (-0.74, 0.84)	-0.28 (-0.99, 0.42)
**Saturated Fat, g**								
Baseline	135	34.6	15.7	143	32.4	13.2	2.57 (-0.75, 5.89)	
Week-18	112	28.1	12.6	121	31	14.1	-2.63 (-6.12, 0.86)	5.20 (2.29, 8.10)
**Saturated Fat, % energy**								
Baseline	135	16.5	4.6	143	16.1	3.9	0.47 (-0.51, 1.45)	
Week-18	112	15.3	3.8	121	16.3	4.3	-0.81 (-1.85, 0.24)	1.28 (0.27, 2.28)
**PUFA, g**								
Baseline	135	13.8	5.9	143	14	5.8	-0.17 (-1.68, 1.34)	
Week-18	112	15.7	8	121	12.8	6.1	2.64 (1.04, 4.24)	-2.81 (-4.26, -1.37)
**PUFA, % energy**								
Baseline	135	6.6	1.7	143	6.9	1.7	-0.37 (-0.80, 0.07)	
Week-18	112	8.4	2.5	121	6.6	1.5	1.72 (1.26, 2.18)	-2.09 (-2.54, -1.63)
**Fibre, g**								
Baseline	135	11.8	3.6	143	12.2	3.6	-0.10 (-2.46, 2.27)	
Week-18	112	24.9	10.6	121	21.2	10.7	3.14 (0.62, 5.65)	-3.24 (-5.61, -0.88)
**Fibre, per 1000 kcal**								
Baseline	135	11.8	3.6	143	12.2	3.6	-0.52 (-1.38, 0.34)	
Week-18	112	15.2	4.3	121	12.2	3.4	2.72 (1.81, 3.63)	-3.24 (-4.07, -2.41)
**Energy Density (g/kcal)**								
Baseline	131	1.95	0.41	143	1.8	0.36	0.16 (0.07, 0.27)	
Week-18	111	1.67	0.38	121	1.87	0.38	-0.18 (-0.27, -0.08)	0.34 (0.24, 0.43)

a PDP minus control diet from linear mixed-effects model.

Supplementary Table 2. Average personalised diet scores in the PDP group over the study months and when stratified across adherence groups.

**Month from plan**	**count**	**mean**	**std**	**min**	**25%**	**50%**	**75%**	**max**
0.00	92.00	61.77	12.11	26.01	55.57	62.76	70.48	85.39
1.00	92.00	62.40	11.82	33.13	54.59	65.02	70.67	83.77
2.00	92.00	63.04	12.47	31.93	57.64	64.14	72.15	87.99
**Adherence groups**								
Low adherence	31.00	49.45	8.36	31.70	42.90	52.69	56.41	59.15
Mid adherence	30.00	63.98	2.60	59.59	62.18	63.60	65.71	68.55
High adherence	31.00	73.83	4.03	68.76	70.99	72.83	75.90	84.45

Supplementary Table 3. Description of primary and secondary outcomes in the METHOD participants by randomized treatment group (ITT, N=347)

	PDP (n=177)						Control (n=170)						Main difference in change between groups		
Primary outcomes	n	Baseline	n	Endpoint	n	Δ baseline -end	n	Baseline	n	Endpoint	n	Δ baseline -end	groups	95% CI *	P-value
LDL-C (mmol/L)	176	3.29 ± 0.78	123	3.32 ± 0.76	123	-0.01 (-0.08, 0.09)	170	3.46 ± 0.88	128	3.46 ± 0.88	128	0.04 (-0.05, 0.13)	-0.04	(-0.16, 0.08)	0.521
**TG (mmol/L)+**	**177**	**1.34 (1.26, 1.42)**	**125**	**1.18 (1.10, 1.26)**	**124**	**-0.22 (-0.32, -0.12)**	**170**	**1.37 (1.29, 1.46)**	**126**	**1.29 (1.21, 1.39)**	**129**	**-0.07 (-0.17, 0.03)**	**-0.13**	**(-0.07, 0.01)**	**0.016**
Secondary outcomes															
**Weight (kg)**	**176**	**93.3 ± 16.9**	**121**	**91.60 ± 19.31**	**121**	**-2.17 (-3.03, -1.31)**	**168**	**95.4 ± 17.5**	**123**	**95.6 ± 18.5**	**121**	**0.30 (-0.56, 1.15)**	**-2.46**	**(-3.67, -1.25)**	**<0.001**
**Waist circumference (cm)**	**176**	**103.5 ± 12.1**	**121**	**101.05 ± 13.55**	**121**	**-2.94 (-4.17, -1.71)**	**169**	**104.0 ± 12.1**	**123**	**103.4 ± 14.0**	**122**	**-0.59 (-1.81, 0.63)**	**-2.35**	**(-4.07, -0.63)**	**0.008**
Hip circumference (cm)	171	114.3 ± 13.5	117	112.80 ± 13.37	114	-1.21 (-2.63, 0.21)	163	116.5 ± 13.0	117	116.3 ± 13.6	114	0.28 (-1.14, 1.70)	-1.49	(-3.48, 0.51)	0.145
Systolic BP (mm/Hg)	176	123.3 ± 16.0	121	121.18 ± 16.71	121	-1.28 (-3.57, 1.00)	168	125.8 ± 16.9	123	124.7 ± 15.8	121	-0.64 (-2.93, 1.64)	-0.64	(-3.87, 2.57)	0.698
Diastolic BP (mm/Hg)	176	79.1 ± 10.6	121	76.50 ± 11.21	121	-1.83 (-3.43, 0.22)	168	80.3 ± 11.1	123	79.5 ± 11.1	121	-0.64 (-2.24, 0.97)	-1.19	(-3.45, 1.06)	0.304
Insulin (mU/L)+	177	8.58 (7.97, 9.23)	102	7.53 (6.82, 8.32)	99	-1.62 (-2.51, -0.72)	170	9.02 (8.40, 9.68)	100	8.32 (7.60, 9.10)	100	-0.98 (-1.87, -0.09)	-0.64	(-3.45, 1.07)	0.304
Glucose (mmol/L)+	177	5.24 (5.16, 5.33)	125	5.17 (5.08, 5.25)	124	-0.02 (-0.09, 0.06)	170	5.40 (5.27, 5.54)	129	5.26 (5.15, 5.38)	129	-0.07 (-0.15, 0.01)	0.06	(-0.01, 0.01)	0.312
**HbA1c (%)+**	**177**	**5.34 (5.30, 5.39)**	**122**	**5.30 (5.25, 5.35)**	**122**	**-0.03 (-0.06, 0.01)**	169	**5.45 (5.36, 5.54)**	**126**	**5.42 (5.34, 5.49)**	**125**	**0.03 (-0.01, 0.06)**	**-0.05**	**(-0.01, -0.001)**	**0.045**
C-peptide	176	1.84 ± 0.79	121	2.25 ± 0.98	121	0.39 (0.31, 0.48)	169	1.9 ± 0.8	125	2.3 ± 0.8	124	0.36 (0.27, 0.45)	0.03	(-0.09, 0.16)	0.591
Apolipoprotein A1 (mg/dL)	176	170.5 ± 29.3	119	165.12 ± 31.64	119	-4.79 (-8.55, -1.03)	169	167.1 ± 29.1	124	164.1 ± 27.8	123	-2.49 (-6.20, 1.22)	-2.3	(-7.55, 2.95)	0.391
Apolipoprotein B (mg/dL)	176	102.2 ± 22.5	119	98.27 ± 20.87	119	-4.32 (-6.70, -1.93)	169	108.3 ± 26.9	124	104.9 ± 24.8	123	-3.82 (-6.17, -1.47)	-0.5	(-3.84, 2.83)	0.769
**Diet quality (HEI, 0-100)**	134	65.6 ± 10.3	110	73.17 ± 8.00	105	**7.01 (5.51, 8.51)**	143	67.9 ± 7.6	120	67.8 ± 8.5	118	**-0.08 (1.35, -1.50)**	**7.08**	**(5.02, 9.15)**	**<0.001**
Shannon Diversity	112	3.9 ± 0.4	112	3.96 ± 0.40	112	0.02 (-0.05, 0.09)	118	3.9 ± 0.4	118	3.9 ± 0.5	118	-0.06 (-0.13, 0.01)	0.08	(-0.01, 0.18)	0.083
Gut species richness	112	212.3 ± 70.3	112	215.24 ± 73.64	112	2.96 (-4.00, 9.91)	118	210.5 ± 65.7	118	213.1 ± 69.0	118	2.58 (-4.20, 9.35)	0.38	(-9.28, 10.0)	0.939

+ Geometric mean and 95% Confidence intervals presented.

* For non-normal variables log-transformed 95% CI on difference in changes between groups are presented

P-values are based on repeated measures analysis with the interaction between the assignment group and time as well as age, and sex as covariates in the model. Primary assessment timepoints include baseline and 18-weeks.

Supplementary Table 4. Description of additional outcomes in the METHOD participants by randomized treatment group (ITT, N=347)

Primary outcomes	***n***	PDP (n=177)<br>Baseline	n	Endpoint	Δ baseline -end	n	Control (n=170)<br>Baseline	n	Endpoint	Δ baseline -end	Main difference in change between groups	95% CI
C-reactive protein	177	3.77 ± 2.97	124	4.04 ± 3.30	0.32 (0.03, 0.60)	170	4.06 ± 2.95	127	4.23 ± 3.28	0.07 (-0.22, 0.35)	0.248	(-0.15, 0.65)
Total cholesterol	177	206.61 ± 32.20	124	202.69 ± 33.84	-4.98 (-9.10, -0.86)	170	211.55 ± 39.25	129	209.81 ± 40.35	-3.30 (-7.36, 0.75)	-1.676	(-7.43, 4.07)
Non-HDL cholesterol	177	145.99 ± 34.17	124	144.13 ± 33.68	-3.32 (-7.13, 0.49)	170	153.52 ± 39.79	129	152.39 ± 37.87	-2.96 (-6.70, 0.79)	-0.363	(-5.68, 4.95)
White blood cell count	176	6.00 ± 1.47	122	5.90 ± 1.71	-0.09 (-0.31, 0.13)	169	6.07 ± 1.55	127	6.19 ± 1.56	0.07 (-0.15, 0.28)	-0.157	(-0.46, 0.15)
Cholesterol to HDL ratio	177	3.61 ± 1.09	124	3.65 ± 1.04	-0.001 (-0.11, 0.10)	170	3.84 ± 1.09	129	3.80 ± 0.96	-0.05 (-0.16, 0.05)	0.051	(-0.10, 0.20)
alkaline phosphatase	176	65.14 ± 17.25	122	65.74 ± 19.52	1.20 (-0.51, 2.92)	169	68.68 ± 20.46	127	69.58 ± 18.95	1.24 (-0.45, 2.92)	-0.033	(-2.43, 2.36)
AST*	176	19.70 ± 10.73	122	18.39 ± 5.51	-1.49 (-2.94, -0.04)	169	18.72 ± 7.85	127	18.65 ± 6.74	0.14 (-1.29, 1.57)	-1.633	(-0.11, 0.02)
ALT*	176	22.67 ± 19.27	122	21.37 ± 14.77	-1.85 (-4.37, 0.66)	169	21.03 ± 13.54	127	21.94 ± 14.34	1.19 (-1.29, 3.67)	-3.042	(-0.17, 0.02)
Bilirubin*	176	0.11 ± 0.04	122	0.11 ± 0.05	0.005 (-0.002, 0.01)	169	0.11 ± 0.03	127	0.11 ± 0.03	0.00 (-0.01, 0.00)	0.007	(-0.002, 0.01)
TNF-alpha*	176	0.75 ± 0.31	98	0.84 ± 0.40	0.06 (0.01, 0.11)	168	0.77 ± 0.30	90	0.78 ± 0.28	-0.01 (-0.06, 0.05)	0.063	(-0.03, 0.17)
Lymphocytes	176	0.32 ± 0.07	122	0.31 ± 0.07	-0.01 (-0.02, 0.00)	169	0.32 ± 0.07	127	0.32 ± 0.08	0.01 (0.00, 0.02)	-0.016	(-0.03, 0.00)
Monocytes	176	0.08 ± 0.02	122	0.08 ± 0.02	0.000 (-0.003, 0.002)	169	0.08 ± 0.02	127	0.07 ± 0.02	-0.002 (0.00, 0.00)	0.001	(-0.002, 0.005)
Neutrophils	176	0.56 ± 0.08	122	0.58 ± 0.08	0.01 (-0.01, 0.02)	169	0.57 ± 0.08	127	0.56 ± 0.09	-0.01 (-0.02, 0.00)	0.016	(-0.001, 0.03)
Albumin globulin	176	1.67 ± 0.29	122	1.67 ± 0.23	0.004 (-0.02, 0.03)	169	1.64 ± 0.24	127	1.64 ± 0.23	-0.007 (-0.03, 0.02)	0.011	(-0.03, 0.05)
Eosinophils*	176	0.03 ± 0.02	122	0.03 ± 0.02	0.000 (-0.003, 0.004)	169	0.03 ± 0.02	127	0.03 ± 0.02	0.002 (-0.002, 0.01)	-0.002	(-0.01, 0.003)
Basophils	176	0.01 ± 0.00	122	0.01 ± 0.00	0.000 (0.00, 0.001)	169	0.01 ± 0.00	127	0.01 ± 0.00	0.004 (-0.035, 0.044)	0.000	(0.000, 0.001)
Globulin b	176	2.61 ± 0.34	122	2.61 ± 0.33	0.002 (-0.04, 0.04)	169	2.64 ± 0.33	127	2.64 ± 0.31	0.004 (-0.03, 0.04)	-0.002	(-0.06, 0.05)
Albumin b	176	4.26 ± 0.23	122	4.30 ± 0.24	0.02 (-0.01, 0.06)	169	4.25 ± 0.24	127	4.25 ± 0.26	-0.01 (-0.05, 0.02)	0.038	(-0.01, 0.09)
Total protein	176	6.87 ± 0.38	122	6.92 ± 0.38	0.03 (-0.03, 0.09)	169	6.89 ± 0.34	127	6.89 ± 0.38	-0.01 (-0.07, 0.05)	0.040	(-0.04, 0.12)
Total bilirubin*	176	0.55 ± 0.23	122	0.57 ± 0.26	0.03 (0.00, 0.06)	169	0.54 ± 0.18	127	0.56 ± 0.20	0.01 (-0.02, 0.04)	0.020	(-0.03, 0.12)
Indirect bilirubin*	176	0.44 ± 0.21	122	0.46 ± 0.23	0.02 (-0.01, 0.05)	169	0.43 ± 0.16	127	0.45 ± 0.18	0.01 (-0.02, 0.04)	0.012	(-0.03, 0.04)
Absolute basophils	176	45.78 ± 20.67	122	48.08 ± 26.64	2.02 (-0.81, 4.86)	169	48.20 ± 20.26	127	49.45 ± 21.56	0.72 (-2.07, 3.52)	1.301	(-2.66, 5.26)
Absolute eosinophils*	176	178.27 ± 167.79	122	163.3 ± 130.7	-6.85 (-34.58, 20.87)	169	175.70 ± 119.34	127	188.00 ± 192.89	14.96 (-12.49, 42.41)	-21.815	(-0.29, 0.02)
RDW	176	0.13 ± 0.01	122	0.13 ± 0.01	0.000 (-0.001, 0.001)	169	0.13 ± 0.01	127	0.13 ± 0.01	0.000 (-0.001, 0.001)	0.000	(-0.001, 0.00)
Haematocrit b	176	0.41 ± 0.03	122	0.42 ± 0.03	0.004 (0.00, 0.008)	169	0.41 ± 0.03	127	0.41 ± 0.03	0.002 (-0.002, 0.01)	0.002	(-0.004, 0.007)
MPV	176	10.33 ± 0.84	122	10.56 ± 0.83	0.21 (0.14, 0.28)	169	10.35 ± 0.88	127	10.45 ± 0.90	0.06 (-0.01, 0.13)	0.148	**(0.05, 0.25)**
MCV	176	89.79 ± 4.38	122	89.22 ± 4.71	-0.64 (-1.01, -0.27)	169	88.50 ± 4.79	127	88.10 ± 4.44	-0.19 (-0.56, 0.17)	-0.450	(-0.97, 0.07)
MCH	176	29.97 ± 1.81	122	29.77 ± 1.86	-0.18 (-0.35, -0.01)	169	29.46 ± 2.04	127	29.33 ± 1.86	-0.09 (-0.26, 0.08)	-0.093	(-0.33, 0.14)
Haemoglobin b	176	13.84 ± 1.09	122	14.01 ± 1.17	0.13 (-0.02, 0.28)	169	13.65 ± 1.13	127	13.70 ± 0.97	0.05 (-0.10, 0.19)	0.084	(-0.12, 0.29)
MCHC	176	33.37 ± 0.80	122	33.37 ± 0.92	0.02 (-0.14, 0.18)	169	33.27 ± 1.05	127	33.28 ± 0.89	-0.01 (-0.17, 0.15)	0.031	(-0.19, 0.25)
Absolute lymphocytes	176	1889 ± 529.62	122	1777 ± 468.5	-74.03 (-130.8, -17.3)	169	1870.54 ± 509.72	127	1957 ± 557.50	75.16 (19.21, 131.10)	-149.2	**(-228.6, -70.0)**
Absolute neutrophils	176	3443 ± 1166	122	3471 ± 1426	6.26 (-186.24, 198.75)	169	3524.98 ± 1244.98	127	3542 ± 1221	-23.00 (-213.28, 167.27)	29.260	(-240.0, 298.8)
Absolute monocytes	176	444.22 ± 115.75	122	438 ± 127	-11.82 (-28.48, 4.85)	169	449.45 ± 126.62	127	449.98 ± 140.13	-0.64 (-17.09, 15.81)	-11.18	(-34.46, 12.15)
Red blood cell count	176	4.63 ± 0.40	122	4.72 ± 0.42	0.07 (0.03, 0.12)	169	4.64 ± 0.38	127	4.68 ± 0.33	0.03 (-0.01, 0.08)	0.043	(-0.02, 0.11)
Platelet count	176	266.72 ± 52.32	122	274 ± 54.7	7.52 (2.08, 12.97)	169	277.77 ± 54.58	127	287.50 ± 56.09	6.58 (1.21, 11.94)	0.948	(-6.66, 8.55)

* For non-normal variables log-transformed 95% CI on difference in changes between groups are presented.

P-values are based on repeated measures analysis with the interaction between the assignment group and time as well as age, and sex as covariates in the model. Primary assessment timepoints include baseline and 18-weeks.

Supplementary Table 5. Median fold change in the bray-Curtis dissimilarity at week-12 and 18 versus baseline.

	Week-12	Week-18
PDP	1.2147	1.3498
Control	1.1980	1.1713
Cross-group comparison	0.9582	1.0890

Supplementary Table 6. Machine learning approach to assess the relationship between change in microbiome composition and change in cardiometabolic health markers across treatment groups

	PDP		Control	
Marker	AUC	Spearman r	AUC	Spearman r
Weight	0.650	0.113	0.490	0.075
Hip circumference	0.591	-0.005	0.490	-0.152
C peptide	0.588	0.069	0.503	0.002
HbA1c	0.552	0.067	0.493	-0.021
Non HDL cholesterol	0.551	-0.002	0.400	0.004
Diet quality (HEI)	0.542	0.208	0.471	0.008
Systolic blood pressure	0.530	-0.072	0.616	-0.021
Diastolic blood pressure	0.526	0.100	0.521	0.212
Apolipoprotein A1	0.508	0.049	0.489	-0.088
Total Cholesterol	0.502	0.032	0.383	-0.065
Insulin	0.500	0.019	0.507	0.092
Triglycerides	0.495	0.245	0.521	0.044
Apolipoprotein B	0.486	-0.094	0.556	0.058
Chol to HDL ratio	0.484	-0.114	0.427	-0.135
Low-density lipoprotein	0.468	-0.059	0.553	0.032
Waist circumference	0.440	0.056	0.590	0.052
Glucose	0.405	0.135	0.509	0.057

Supplementary Table 7. Relative abundances of 30 microbial species and change in the PDP and control groups.

	PDP					Control						Median fold change	
Primary outcomes	***n***	Baseline	n	Endpoint	Δ end-baseline	n	Baseline	n	Endpoint	Δ end-baseline	P-value	PDP	Control
Favourable species													
Firmicutes_bacterium_CAG_95	112	0.01 ± 0.04	112	0.01 ± 0.07	0.01 ± 0.05	117	0.01 ± 0.04	117	0.01 ± 0.07	0.00 ± 0.07	0.08	1.5797	1.4832
Haemophilus_parainfluenzae	112	0.01 ± 0.02	112	0.00 ± 0.01	0.00 ± 0.02	117	0.01 ± 0.03	117	0.00 ± 0.01	0.00 ± 0.03	**0.05**	0.4363	0.4543
Oscillibacter_sp_57_20	112	0.42 ± 0.58	112	0.60 ± 0.80	0.18 ± 0.60	117	0.48 ± 0.66	117	0.46 ± 0.74	-0.02 ± 0.63	**0.01**	1.4304	0.9674
Firmicutes_bacterium_CAG_170	112	0.00 ± 0.01	112	0.00 ± 0.01	0.00 ± 0.00	117	0.01 ± 0.07	117	0.01 ± 0.04	0.00 ± 0.06	0.63	1.6570	0.7121
Roseburia_sp_CAG_182	112	0.03 ± 0.18	112	0.12 ± 0.77	0.10 ± 0.78	117	0.02 ± 0.09	117	0.01 ± 0.04	-0.01 ± 0.09	**0.02**	4.8021	0.6478
Clostridium_sp_CAG_167	112	0.16 ± 0.63	112	0.26 ± 0.83	0.10 ± 0.84	117	0.12 ± 0.46	117	0.12 ± 0.39	0.00 ± 0.44	0.05	1.6039	0.9609
Oscillibacter_sp_PC13	112	0.00 ± 0.00	112	0.00 ± 0.00	0.00 ± 0.00	117	0.00 ± 0.00	117	0.00 ± 0.00	0.00 ± 0.00	0.72	0.6724	0.7768
Eubacterium_eligens	112	0.90 ± 1.27	112	1.42 ± 1.99	0.52 ± 1.62	117	1.36 ± 1.81	117	1.29 ± 1.84	-0.07 ± 1.91	**0.01**	1.5720	0.9493
Prevotella_copri	112	4.26 ± 10.76	112	2.97 ± 8.41	-1.29 ± 7.06	117	4.58 ± 11.22	117	4.17 ± 10.62	-0.41 ± 8.18	0.45	0.6975	0.9103
Veillonella_dispar	112	0.00 ± 0.01	112	0.01 ± 0.08	0.01 ± 0.08	117	0.00 ± 0.01	117	0.00 ± 0.02	0.00 ± 0.02	0.80	5.9661	1.4867
Faecalibacterium_prausnitzii	112	6.26 ± 4.66	112	7.08 ± 4.83	0.82 ± 4.48	117	6.77 ± 4.68	117	6.58 ± 4.68	-0.19 ± 3.88	**0.03**	1.1316	0.9723
Veillonella_infantium	112	0.00 ± 0.01	112	0.01 ± 0.05	0.00 ± 0.05	117	0.00 ± 0.00	117	0.00 ± 0.01	0.00 ± 0.01	0.42	5.4241	2.5051
Bifidobacterium_animalis	112	0.03 ± 0.11	112	0.06 ± 0.17	0.03 ± 0.20	117	0.07 ± 0.48	117	0.04 ± 0.19	-0.03 ± 0.52	0.08	2.2248	0.6024
Romboutsia_ilealis	112	0.01 ± 0.01	112	0.01 ± 0.01	0.00 ± 0.02	117	0.01 ± 0.02	117	0.01 ± 0.01	0.00 ± 0.02	0.39	0.8674	0.7518
Veillonella_atypica	112	0.01 ± 0.03	112	0.01 ± 0.03	0.00 ± 0.02	117	0.00 ± 0.01	117	0.00 ± 0.03	0.00 ± 0.03	0.95	1.1206	1.0988
Unfavourbale species													
Clostridium_leptum	112	0.15 ± 0.52	112	0.09 ± 0.24	-0.06 ± 0.55	117	0.12 ± 0.35	117	0.12 ± 0.34	0.00 ± 0.48	0.11	0.5917	1.0196
Ruthenibacterium_lactatiformans	112	0.22 ± 0.47	112	0.39 ± 1.45	0.17 ± 1.48	117	0.22 ± 0.35	117	0.36 ± 0.77	0.13 ± 0.74	0.64	1.7635	1.6002
Collinsella_intestinalis	112	0.24 ± 0.97	112	0.28 ± 1.15	0.04 ± 1.02	117	0.36 ± 1.27	117	0.39 ± 1.30	0.03 ± 0.63	0.44	1.1622	1.0742
Escherichia_coli	112	0.17 ± 1.15	112	0.25 ± 1.17	0.08 ± 0.51	117	0.16 ± 0.73	117	0.34 ± 1.24	0.18 ± 1.00	0.17	1.4827	2.1121
Blautia_hydrogenotrophica	112	0.11 ± 0.21	112	0.14 ± 0.29	0.03 ± 0.30	117	0.11 ± 0.31	117	0.10 ± 0.20	-0.01 ± 0.27	0.89	1.3070	0.8748
Eggerthella_lenta	112	0.30 ± 0.55	112	0.42 ± 0.67	0.11 ± 0.52	117	0.30 ± 0.43	117	0.29 ± 0.42	-0.01 ± 0.32	0.08	1.3740	0.9630
Clostridium_sp_CAG_58	112	0.28 ± 0.36	112	0.23 ± 0.24	-0.05 ± 0.34	117	0.25 ± 0.35	117	0.24 ± 0.36	-0.01 ± 0.29	0.85	0.8145	0.9597
Ruminococcus_gnavus	112	1.17 ± 2.50	112	0.91 ± 2.41	-0.26 ± 2.09	117	0.82 ± 1.69	117	0.93 ± 2.59	0.11 ± 2.25	0.06	0.7806	1.1301
Clostridium_spiroforme	112	0.05 ± 0.10	112	0.08 ± 0.15	0.03 ± 0.13	117	0.06 ± 0.10	117	0.07 ± 0.12	0.01 ± 0.09	0.28	1.5939	1.2068
Clostridium_bolteae_CAG_59	112	0.01 ± 0.04	112	0.01 ± 0.03	0.00 ± 0.03	117	0.02 ± 0.12	117	0.04 ± 0.26	0.01 ± 0.27	0.52	0.8064	1.5932
Clostridium_innocuum	112	0.05 ± 0.13	112	0.09 ± 0.29	0.04 ± 0.21	117	0.05 ± 0.13	117	0.04 ± 0.10	-0.01 ± 0.12	0.36	1.7123	0.8709
Anaerotruncus_colihominis	112	0.02 ± 0.05	112	0.03 ± 0.06	0.00 ± 0.05	117	0.01 ± 0.02	117	0.02 ± 0.04	0.01 ± 0.04	0.80	1.0733	1.5048
Clostridium_symbiosum	112	0.05 ± 0.18	112	0.05 ± 0.18	0.01 ± 0.13	117	0.02 ± 0.05	117	0.02 ± 0.06	0.00 ± 0.06	0.31	1.1386	1.0147
Clostridium_bolteae	112	0.05 ± 0.11	112	0.05 ± 0.11	0.00 ± 0.10	117	0.05 ± 0.12	117	0.09 ± 0.38	0.04 ± 0.38	**0.04**	1.0063	1.6847
Flavonifractor_plautii	112	0.53 ± 1.13	112	0.49 ± 0.80	-0.04 ± 0.96	117	0.42 ± 0.80	117	0.44 ± 0.71	0.02 ± 0.50	0.45	0.9238	1.0585
Favourable species (sum)	112	12.09 ± 12.13	112	12.57 ±	0.48 ± 9.05	117	13.44 ±	117	12.71 ±	-0.73 ± 8.63	**0.02**		
Unfavourable species (sum)	112	3.41 ± 4.35	112	3.51 ± 5.02	0.01 ± 3.67	117	2.98 ± 4.03	117	3.48 ± 4.38	0.50 ± 3.43	0.67		

P-value was calculated using Mann-Whitney-Wilcoxon Test.
Mean ± SD presented for all.

Supplementary Table 8. Description of cardiometabolic health measures and changes after the personalised diet and control diet (Per protocol analysis, N=225)

		PDP (n=108)					Control (n=117)						
Primary outcomes	n	Baseline	n	Endpoint	Δ baseline -end	n	Baseline	n	Endpoint	Δ baseline -end	Main difference in change between groups	95% CI*	P-value
LDL-C (mmol/L)	108	3.32 ± 0.72	108	3.32 ± 0.77	0.00 (-0.09, 0.10)	117	3.52 ± 0.88	117	3.46 ± 0.87	-0.05 (-0.14, 0.04)	0.05	(-0.08, 0.19)	0.430
**TG (mmol/L)**	**108**	**1.38 (1.28, 1.48)**	**108**	**1.20 (1.11, 1.29)**	**-0.23 (-0.33, -0.12)**	**117**	**1.32 (1.23, 1.41)**	**117**	**1.25 (1.17, 1.34)**	**-0.06 (-0.16, 0.05)**	**-0.17**	**(-0.07, -0.01)**	**0.032**
Secondary outcomes													
**Weight (kg)**	**108**	**92.79 ± 17.38**	**107**	**90.28 ± 18.51**	**-2.42 (-3.35, -1.51)**	**116**	**94.99 ± 17.32**	**113**	**94.54 ± 17.54**	**0.08 (-0.82, 0.98)**	**-2.51**	**(-3.79, -1.23)**	**<0.001**
**Waist circumference (cm)**	**108**	**103.58 ± 12.16**	**107**	**100.53 ± 13.34**	**-3.04 (-4.39, -1.69)**	**117**	**103.55 ± 12.02**	**113**	**102.41 ± 13.28**	**-0.91 (-2.22, 0.40)**	**-2.13**	**(4.00, -0.26)**	**0.027**
Hip circumference (cm)	105	113.71 ± 13.90	104	112.61 ± 13.30	-1.07 (-2.42, 0.29)	115	116.02 ± 13.71	107	115.46 ± 13.32	0.18 (-1.55, 1.91)	-1.13	(-3.30, 1.05)	0.310
Systolic BP (mm/Hg)	108	122.14 ± 15.40	107	121.05 ± 16.93	-1.11 (-3.59, 1.38)	116	124.62 ± 15.80	113	124.20 ± 15.17	-0.52 (-2.94, 1.91)	-0.59	(-4.04, 2.87)	0.740
Diastolic BP (mm/Hg)	108	77.91 ± 10.26	107	76.32 ± 11.06	-1.57 (-3.28, 0.15)	116	79.73 ± 10.40	113	78.98 ± 10.88	-0.68 (-2.35, 1.00)	-0.89	(-3.27, 1.50)	0.466
Insulin (mU/L)+	108	8.61 (7.85, 9.46)	88	7.25 (6.59, 7.89)	-1.83 (-2.74, -0.91)	117	8.87 (8.17, 9.62)	91	7.90 (7.27, 8.70)	-0.93 (-1.83, 0.04)	-0.89	(-0.09, 0.02)	0.193
Glucose (mmol/L)+	108	5.20 (5.11, 5.30)	108	5.16 (5.07, 5.26)	-0.04 (-0.12, 0.05)	117	5.27 (5.18, 5.37)	117	5.21 (5.11, 5.32)	-0.05 (-0.14, 0.03)	0.01	(-0.01, 0.01)	0.738
HbA1c (%) +	108	5.32 (5.27, 5.37)	106	5.30 (5.25, 5.35)	-0.02 (-0.05, 0.01)	116	5.38 (5.32, 5.43)	116	5.38 (5.33, 5.45)	0.01 (-0.02, 0.04)	-0.03	(-0.01, 0.001)	0.152
C-peptide	108	1.85 ± 0.82	106	2.23 ± 1.00	0.37 (0.27, 0.46)	116	1.85 ± 0.63	115	2.21 ± 0.76	0.37 (0.28, 0.47)	-0.01	(-0.14, 0.13)	0.930
Apolipoprotein A1 (mg/dL)	108	169.08 ± 30.39	105	163.93 ± 30.86	-4.88 (-8.70, -0.96)	116	167.50 ± 28.69	115	164.28 ± 27.85	-2.78 (-6.55, 0.97)	-2.09	(-7.49, 3.31)	0.449
Apolipoprotein B (mg/dL)	108	103.93 ± 22.21	105	98.61 ± 21.15	-4.76 (-7.28, -2.25)	116	107.95 ± 25.62	115	104.24 ± 24.14	-3.59 (-6.01, -1.18)	-1.17	(-4.64, 2.29)	0.509
**Diet quality (HEI, 0-100)**	**102**	**66.40 ± 10.55**	**101**	**73.50 ± 7.91**	**6.99 (5.37, 8.62)**	**114**	**68.26 ± 7.47**	**108**	**67.91 ± 8.54**	**0.32 (-1.23, 1.87)**	**7.32**	**(5.08, 9.55)**	**<0.001**
Shannon diversity	99	3.90 ± 0.40	99	3.96 ± 0.38	0.04 (-0.03, 0.11)	109	3.90 ± 0.39	109	3.87 ± 0.46	-0.05 (-0.12, 0.01)	0.09	(0.02, 0.17)	0.052
Gut species richness	99	211.45 ± 70.90	99	215.99 ± 70.31	4.54 (-2.52, 11.6)	109	211.16 ± 66.65	109	212.21 ± 70.20	1.06 (-5.67, 7.78)	3.48	(-6.21, 13.2)	0.482

+ Geometric mean and 95% Confidence intervals presented.

* For non-normal variables log-transformed 95% CI on difference in changes between groups are presented.

P-value based on mixed regression model with an interaction between assignment group and time as well as age, and sex as covariates.

Supplementary Table 9. Stratification by adherence across treatments

	Low adherence PDP participants		High adherence PDP participants		High vs Low PDP	High adherence control participants		High PDP vs High control
	**n**	**change (SD)**	**n**	**change (SD)**	P-value*	**n**	**change (SD)**	P-value*
Triglycerides (mmol/L)	33	-0.29 (0.75)	35	-0.20 (0.38)	0.75	39	0.02 (0.53)	0.062
LDL-C (mmol/L)	32	0.07 (0.56)	35	-0.20 (0.48)	**0.019**	39	-0.10 (0.51)	0.360
Secondary outcomes								
Weight (kg)	33	-2.01 (7.78)	32	-4.09 (4.51)	0.139	34	-0.44 (3.27)	**0.002**
Waist circumference (cm)	33	-1.42 (5.95)	31	-6.31 (5.35)	**0.001**	35	-3.30 (9.42)	0.099
Hip circumference (cm)	29	-1.36 (6.87)	30	-0.68 (8.0)	0.959	30	-0.55 (9.05)	0.982
Diastolic BP (mm/Hg)	33	2.71 (9.23)	31	-4.08 (8.56)	**0.002**	34	-1.32 (8.39)	0.166
Systolic BP (mm/Hg)	33	3.00 (11.16)	31	-1.23 (14.38)	0.252	34	-0.78 (14.74)	0.977
Glucose (mmol/L)	33	0.61 (6.98)	35	-0.73 (7.94)	0.44	39	-0.44 (8.3)	0.908
HbA1c (%)	33	0.02 (0.12)	34	-0.06 (0.2)	**0.024**	38	0.02 (0.19)	0.068
Insulin (mU/L)	23	-0.8 (5.23)	34	-1.97 (3.35)	0.181	31	-1.39 (5.57)	0.574
C-peptide	32	0.48 (0.46)	34	0.26 (0.55)	0.075	37	0.3 (0.35)	0.712
Apolipoprotein B (mg/dL)	32	-5.56 (11.28)	34	-7.94 (13.7)	0.345	37	-1.14 (12.84)	**0.025**
Apolipoprotein A1 (mg/dL)	32	3.39 (15.62)	34	-12.7 (26.19)	**0.001**	37	-7.22 (17.18)	0.357
Gut species richness	31	7.68 (22.31)	33	5.15 (45.16)	0.769	39	1.1 (32.77)	0.660
Gut diversity (Shannon)	31	0.06 (0.22)	33	0.07 (0.32)	0.786	39	-0.04 (0.22)	0.063
Non-HDL cholesterol (mmol/L)	33	-0.07 (0.52)	35	-0.27 (0.52)	0.093	39	-0.07 (0.58)	0.119
Cholesterol-HDL ratio	33	-0.14 (0.4)	35	-0.06 (0.56)	0.533	39	0.06 (0.54)	0.335
Total cholesterol (mmol/L)	33	-0.01 (0.58)	35	-0.4 (0.51)	**0.002**	39	-0.13 (0.63)	**0.047**
Post-prandial TG (6h conc.)	25	0.23 (1.07)	34	-0.08 (0.92)	0.189			
Diet quality (HEI)	30	4.26 (8.71)	33	10.15 (9.15)	**0.01**	40	4.0 (6.59)	**0.000**
	**n**	**% users improving**	**n**	**% users improving**				
Energy	30	60	35	71.42	0.492	-	-	-
Hunger	30	66.67	35	88.57	**0.015**	-	-	-
Mood	30	50	35	54.29	0.673	-	-	-
Skin quality	30	16.67	35	22.86	0.323	-	-	-

Adherence is based on 33ntile comparison (i.e. low adherence is the bottom 33 percentile of adherence score and high adherence is the top 33ntile). High adherence; participants with average day score > 68. Low adherence; participants with average day score < 59. P-value from the mixed model regression analysis. Energy, hunger, mood and skin quality reported as a categorial answer; No, I don’t agree/I have experienced no change/Yes, I Agree.

Supplementary Table 10. Outcome measures assessed in the ZOE METHOD study

Outcome	Units	Source	Baseline	Midpoint	Endpoint
Clinical Blood Chemistry					
Triglycerides	mmol/L	Venous blood draw (fasted); DBS (postprandial)	X	X	X
Total Cholesterol	mmol/L	Venous blood draw (fasted); DBS (postprandial)	X	X	X
Direct LDL-cholesterol	mmol/L	Venous blood draw (fasted)	X	X	X
Calculated LDL-cholesterol	mmol/L	Venous blood draw (fasted)	X	X	X
HDL-cholesterol	mmol/L	Venous blood draw (fasted); DBS (postprandial)	X	X	X
Cholesterol/HDL-cholesterol ratio	-	Venous blood draw (fasted)	X	X	X
non-HDL-cholesterol	mmol/L	Venous blood draw (fasted)	X	X	X
Apolipoprotein A1	mg/dL	Venous blood draw (fasted)	X		X
Apolipoprotein B	mg/dL	Venous blood draw (fasted)	X		X
Glucose	mmol/L	Venous blood draw (fasted); continuous (CGM)	X	X	X
C-peptide	ng/mL	Venous blood draw (fasted)	X		X
Intact Insulin	uIU/mL	Venous blood draw (fasted)	X	X	X
HbA1c	%	Venous blood draw (fasted)	X		X
hsCRP	mg/L	Venous blood draw (fasted)	X	X	X
Total Protein	g/dL	Venous blood draw (fasted)	X		X
Albumin	g/dL	Venous blood draw (fasted)	X		X
Globulin	g/dL	Venous blood draw (fasted)	X		X
Albumin/Globulin ratio	-	Venous blood draw (fasted)	X		X
Total bilirubin	mg/dL	Venous blood draw (fasted)	X		X
Direct bilirubin	mg/dL	Venous blood draw (fasted)	X		X
Indirect bilirubin	mg/dL	Venous blood draw (fasted)	X		X
Alkaline phosphatase	U/L	Venous blood draw (fasted)	X		X
Aspartate transferase	U/L	Venous blood draw (fasted)	X		X
Alanine transaminase	U/L	Venous blood draw (fasted)	X		X
hsTNF-alpha	pg/mL	Venous blood draw (fasted)	X		X
White blood cell count	Thousand/uL	Venous blood draw (fasted)	X		X
Red blood cell count	Million/uL	Venous blood draw (fasted)	X		X
Haemoglobin	g/dL	Venous blood draw (fasted)	X		X
Haematocrit	%	Venous blood draw (fasted)	X		X
Mean corpuscular volume	fL	Venous blood draw (fasted)	X		X
Mean corpuscular haemoglobin	pg	Venous blood draw (fasted)	X		X
Mean corpuscular haemoglobin	g/dL	Venous blood draw (fasted)	X		X
Red blood cell distribution width	%	Venous blood draw (fasted)	X		X
Platelet count	Thousand/uL	Venous blood draw (fasted)	X		X
Mean platelet volume	fL	Venous blood draw (fasted)	X		X
Absolute Neutrophils	cells/UL	Venous blood draw (fasted)	X		X

Absolute Lymphocytes	cells/UL	Venous blood draw (fasted)	X		X
Absolute Monocytes	cells/UL	Venous blood draw (fasted)	X		X
Absolute Eosinophils	cells/UL	Venous blood draw (fasted)	X		X
Absolute Basophils	cells/UL	Venous blood draw (fasted)	X		X
Neutrophils	%	Venous blood draw (fasted)	X		X
Lymphocytes	%	Venous blood draw (fasted)	X		X
Monocytes	%	Venous blood draw (fasted)	X		X
Eosinophils	%	Venous blood draw (fasted)	X		X
Basophils	%	Venous blood draw (fasted)	X		X
Biometrics					
Body weight	lbs	Clinical measure	X	X	X
Height	ft, in	Clinical measure	X	X	X
Waist circumference	in	Clinical measure	X	X	X
Hip circumference	in	Clinical measure	X	X	X
Systolic blood pressure	mmHg	Clinical measure	X	X	X
Diastolic blood pressure	mmHg	Clinical measure	X	X	X
Dietary intake					
Habitual food and nutrient intake	g / day	Food Frequency Questionnaire	X	X	X
Acute food and nutrient intake	g / day	Weighed food diary	X	X	X
Gut Microbiome					
Species richness	no of species	Stool sample	X	X	X
Shannon diversity		Stool sample	X	X	X
Beta-diversity (Bray Curtis) dissimilarity		Stool sample	X	X	X
Subjective Data					
Hunger	nominal	Survey	X	X	X
Mood	nominal	Survey	X	X	X
Energy	nominal	Survey	X	X	X
Sleep	nominal	Survey	X	X	X
Skin quality	nominal	Survey	X	X	X
Menopausal symptom burden	nominal	Survey	X	X	X
Adherence	nominal	Survey	X	X	X

LDL, low-density lipoprotein; HDL, high-density lipoprotein; HbA1c, glycated haemoglobin; hsCRP, high-sensitivity c-reactive protein; hsTNF, high-sensitivity tumour necrosis factor; mmol, millimole; L, litre; mg, milligrams; dL, decilitre; ng, nanogram; IU, international unit; %, percentage; g, grams; fL, fluid ounces; pg, picograms; lbs, pounds; ft, feet; in, inches; mmHg, millimetre of mercury; DBS, dried-blood spot; CGM, continuous glucose monitor.
