# Supplementary material for: Effects of a personalized nutrition program on cardiometabolic health: a randomized controlled trial
Source: Nat Med. 2024 May 8;30(7):1888–97. doi: 10.1038/s41591-024-02951-6 (PMC11271409; doi:10.1038/s41591-024-02951-6)
Supplement: Supplementary file 2 — Reporting Summary [file 41591_2024_2951_MOESM2_ESM.pdf]

nature portfolio

Corresponding author(s): Dr Sarah Berry

Last updated by author(s): October 3rd 2023

Reporting Summary

Nature Portfolio wishes to improve the reproducibility of the work that we publish. This form provides structure for consistency and transparency in reporting. For further information on Nature Portfolio policies, see our Editorial Policies and the Editorial Policy Checklist.

Statistics

For all statistical analyses, confirm that the following items are present in the figure legend, table legend, main text, or Methods section.

n/a	Confirmed	
☐	☒	The exact sample size (*n*) for each experimental group/condition, given as a discrete number and unit of measurement
☐	☒	A statement on whether measurements were taken from distinct samples or whether the same sample was measured repeatedly
☐	☒	The statistical test(s) used AND whether they are one- or two-sided<br>*Only common tests should be described solely by name; describe more complex techniques in the Methods section.*
☐	☒	A description of all covariates tested
☐	☒	A description of any assumptions or corrections, such as tests of normality and adjustment for multiple comparisons
☐	☒	A full description of the statistical parameters including central tendency (e.g. means) or other basic estimates (e.g. regression coefficient) AND variation (e.g. standard deviation) or associated estimates of uncertainty (e.g. confidence intervals)
☐	☒	For null hypothesis testing, the test statistic (e.g. *F*, *t*, *r*) with confidence intervals, effect sizes, degrees of freedom and *P* value noted<br>*Give P values as exact values whenever suitable.*
☒	☐	For Bayesian analysis, information on the choice of priors and Markov chain Monte Carlo settings
☒	☐	For hierarchical and complex designs, identification of the appropriate level for tests and full reporting of outcomes
☒	☐	Estimates of effect sizes (e.g. Cohen's *d*, Pearson's *r*), indicating how they were calculated

Our web collection on statistics for biologists contains articles on many of the points above.

Software and code

Policy information about availability of computer code

Data collection	Data from questionnaires, clinical visits and laboratory data was entered using comma delimited files, Excel spreadsheets (Version 16.82) and Microsoft access (Microsoft Office 365 2019)
Data analysis	Analyses were carried out using version 4.0.2 R Core Team and Python 3.9.7. Pandas 1.1.3, numpy 1.23.5 and scipy 1.11.1 were used to manage and preprocess data.<br>Code availability statement: The scripts for statistical analysis are freely available upon request to ZOE Ltd. Application is via data.papers@joinzoe.com. Code will be made available within 2 months of request.

For manuscripts utilizing custom algorithms or software that are central to the research but not yet described in published literature, software must be made available to editors and reviewers. We strongly encourage code deposition in a community repository (e.g. GitHub). See the Nature Portfolio guidelines for submitting code & software for further information.

Data

Policy information about availability of data

All manuscripts must include a data availability statement. This statement should provide the following information, where applicable:

- Accession codes, unique identifiers, or web links for publicly available datasets
- A description of any restrictions on data availability
- For clinical datasets or third party data, please ensure that the statement adheres to our policy

The data can be released to bona fide researchers submitting a research proposal approved by a sub-panel of our Scientific Advisory Board. We have meetings once per month with independent members to assess proposals. The data will be anonymised and conform to UK GDPR standards. Access request proposals should be sent to data.papers@joinzoe.com. The microbiome data will be uploaded onto the EBI website (https://www.ebi.ac.uk/).

Research involving human participants, their data, or biological material

Policy information about studies with human participants or human data. See also policy information about sex, gender (identity/presentation), and sexual orientation and race, ethnicity and racism.

Reporting on sex and gender	The study included males and females, of which 86% of the participants were female. We do not report any stratified analysis based on sex or gender.
Reporting on race, ethnicity, or other socially relevant groupings	The study included multiple ethnicities, of which 82% were white. We do not report any stratified analysis based on race, ethnicity or other socially relevant groupings.
Population characteristics	Study participants were healthy individuals, including males and females reflective of the average US adult population [aged 40–70 y; waist circumference greater than ethnic- and sex-specific 25th percentile values; fruit and vegetable intake below 450g/day].
Recruitment	Participants were recruited (March 2022–August 2022) by electronic advertisement (e-mail to the Stanford Nutrition Research Studies Cohort, the Empowered Gut newsletter, and ZOE Ltd mailing lists).
Ethics oversight	Ethics was granted by Advarra Institutional Review Board (IRB no. 00000971; Protocol Number. 00044316).

Note that full information on the approval of the study protocol must also be provided in the manuscript.

Field-specific reporting

Please select the one below that is the best fit for your research. If you are not sure, read the appropriate sections before making your selection.

☒ Life sciences ☐ Behavioural & social sciences ☐ Ecological, evolutionary & environmental sciences

For a reference copy of the document with all sections, see nature.com/documents/nr-reporting-summary-flat.pdf

Life sciences study design

All studies must disclose on these points even when the disclosure is negative.

Sample size	347 individuals were recruited for the US cohort (powered on a sample size of 150 participants per group (N=300) at 90% power and P < 0.05, to detect a 0.21 mmol/L between group difference in triglycerides).
Data exclusions	Pre-established exclusion criteria are listed on ClincialTrials.gov
Replication	Given the randomised controlled trial design, replication was not applicable.
Randomization	Participants were randomly and equally allocated to one of the two treatments based on the following minimisation factors: (i) sex, male or female; (ii) waist circumference, above or below their ethnic-specific median; (iii) fruit and vegetable intake, above or below the median US adult intake of 234 g per day.
Blinding	A blinded researcher performed the between group analysis. Group allocation was concealed by labelling the groups with non-identifying terms.

Reporting for specific materials, systems and methods

We require information from authors about some types of materials, experimental systems and methods used in many studies. Here, indicate whether each material, system or method listed is relevant to your study. If you are not sure if a list item applies to your research, read the appropriate section before selecting a response.

Materials & experimental systems

n/a	Involved in the study
☒	☐ Antibodies
☒	☐ Eukaryotic cell lines
☒	☐ Palaeontology and archaeology
☒	☐ Animals and other organisms
☐	☒ Clinical data
☒	☐ Dual use research of concern
☒	☐ Plants

Methods

n/a	Involved in the study
☒	☐ ChIP-seq
☒	☐ Flow cytometry
☒	☐ MRI-based neuroimaging

Clinical data

Policy information about clinical studies

All manuscripts should comply with the ICMJE guidelines for publication of clinical research and a completed CONSORT checklist must be included with all submissions.

Clinical trial registration	NCT05273268
Study protocol	https://clinicaltrials.gov/study/NCT05273268
Data collection	Participants were recruited between 1 March 2022 – 10 August 2022. Data were collected remotely and at Quest Diagnostic Patient Service Centers, USA.
Outcomes	Primary outcomes were serum triglyceride (TG) and direct low-density lipoprotein cholesterol (LDL-C) concentrations, secondary outcomes were changes in weight, waist circumference, hip circumference, blood pressure (BP) (systolic and diastolic), blood haemoglobin A1C (HbA1c), serum insulin, serum glucose, serum C-peptide, serum apolipoprotein A1, serum apolipoprotein B, faecal gut microbiome (species richness, Shannon's diversity and Bray-Curtis dissimilarity), postprandial blood TG concentrations, habitual diet quality (Healthy Eating Index), and self-reported energy level. Bloods were measured at Quest Diagnostic Patient Service Centers, USA, all other outcomes were measured remotely.

Plants

Seed stocks	*Report on the source of all seed stocks or other plant material used. If applicable, state the seed stock centre and catalogue number. If plant specimens were collected from the field, describe the collection location, date and sampling procedures.*
Novel plant genotypes	*Describe the methods by which all novel plant genotypes were produced. This includes those generated by transgenic approaches, gene editing, chemical/radiation-based mutagenesis and hybridization. For transgenic lines, describe the transformation method, the number of independent lines analyzed and the generation upon which experiments were performed. For gene-edited lines, describe the editor used, the endogenous sequence targeted for editing, the targeting guide RNA sequence (if applicable) and how the editor was applied.*
Authentication	*Describe any authentication procedures for each seed stock used or novel genotype generated. Describe any experiments used to assess the effect of a mutation and, where applicable, how potential secondary effects (e.g. second site T-DNA insertions, mosiacism, off-target gene editing) were examined.*
